# Creation of consensus recommendations for collaborative practice in the Malaysian psychiatric system: a modified Delphi study

**DOI:** 10.1186/s13033-020-00374-7

**Published:** 2020-06-19

**Authors:** Wendy Shoesmith, Sze Hung Chua, Beena Giridharan, Dawn Forman, Sue Fyfe

**Affiliations:** 1 Faculty of Medicine and Health Sciences, University Malaysia Sabah, Kota Kinabalu, Malaysia; 2 Curtin University, Miri, Sarawak Malaysia; 3 Hospital Mesra Bukit Padang, Ministry of Health, Kota Kinabalu, Malaysia; 4 School of Public Health, Curtin University, Perth, Australia; 5 University of Derby, Derby, UK

**Keywords:** Collaborative practice, Delphi method, Consensus methods, Malaysia, Mental health, Guidelines, User participation

## Abstract

**Background:**

There is strong evidence that collaborative practice in mental healthcare improves outcomes for patients. The concept of collaborative practice can include collaboration between healthcare workers of different professional backgrounds and collaboration with patients, families and communities. Most models of collaborative practice were developed in Western and high-income countries and are not easily translatable to settings which are culturally diverse and lower in resources. This project aimed to develop a set of recommendations to improve collaborative practice in Malaysia.

**Methods:**

In the first phase, qualitative research was conducted to better understand collaboration in a psychiatric hospital (previously published). In the second phase a local hospital level committee from the same hospital was created to act on the qualitative research and create a set of recommendations to improve collaborative practice at the hospital for the hospital. Some of these recommendations were implemented, where feasible and the outcomes discussed. These recommendations were then sent to a nationwide Delphi panel. These committees consisted of healthcare staff of various professions, patients and carers.

**Results:**

The Delphi panel reached consensus after three rounds. The recommendations include ways to improve collaborative problem solving and decision making in the hospital, ways to improve the autonomy and relatedness of patients, carers and staff and ways to improve the levels of resources (e.g. skills training in staff, allowing people with lived experience of mental disorder to contribute).

**Conclusions:**

This study showed that the Delphi method is a feasible method of developing recommendations and guidelines in Malaysia and allowed a wider range of stakeholders to contribute than traditional methods of developing guidelines and recommendations.

*Trial registration* Registered in the National Medical Research Register, Malaysia, NMRR-13-308-14792

## Background

Collaborative practice is defined by the World Health Organisation as: “when multiple health workers from different professional backgrounds work together with patients, families, carers and communities to deliver the highest quality of care” [1]. Collaborative practice is a broad term, which includes collaboration between the patient and healthcare staff, collaboration between the different members of the multidisciplinary team, collaboration between primary care and mental health staff and collaboration between healthcare staff and other members of the community. The complexity of severe mental problems mean that the good care is normally team based, with several different professions working together to help the patient. In the care of people with mental health problems, collaboration between service users and healthcare providers allows them to work towards common goals and this partnership has been shown to be one of the most efficacious components of many treatments [2–6]. Evidence that some of these collaborative models are more effective than usual care is strong, particularly collaborations between primary care and mental health services, where more than 80 randomised controlled trials have demonstrated clear improvements in outcomes with no increase in costs [7].

Developing collaborative practice in Malaysia was considered important because qualitative research showed that interactions were often hierarchical, rather than collaborative [8]. This was sometimes having a negative effect on patient care, for example nurses not telling doctors if they believed a treatment plan would not work and patients not discussing with their doctors if they had stopped medication due to side effects. Service provision was often siloed, for example there was little communication between the psychiatric hospital and community health clinics [8, 9]. There was also a large treatment gap of more than 90% in primary care [10]. Most collaborative models of care were developed in Western cultures. Cultural factors affect the way that people work together, and a model of care developed in a Western setting may not be the best model of care for an Asian context. These models are complex, with many elements and it is difficult to know what the most important elements for effectiveness are.

Developing complex models of care and interventions requires a different approach to developing simple interventions. Consensus methods offer a way of creating guidelines where the proposed intervention is complex and in situations where there is no strong evidence [11, 12]. Consensus methods include Delphi methods, nominal group techniques and the consensus development conference [12]. Delphi methods have been used extensively in higher-income countries and some low-income countries as a way of improving mental health services [13, 14] as well as a way of developing culturally appropriate responses [15]. The Delphi method involves sending a panel a series of items to rate and comment on. The panel never meet in person and all their ratings and comments remain anonymous. After the first round, the panel are sent the ratings and comments of the other panel members and the process is repeated in a series of rounds until consensus is reached [16]. In this study the Delphi method was chosen as a way of generalising recommendations developed by a local hospital level committee so that they would be useful for the whole of Malaysia. The Delphi method was chosen because it has been shown to be a reliable way of reaching a consensus [17], the anonymity makes it easier for people to express ideas if there is any perceived hierarchy and because we wished to get opinions from different geographical areas of Malaysia, where face-to-face meetings can be difficult and expensive.

This exercise aimed to answer the question: ‘What will improve collaboration in the Malaysian Mental Health System?’ The aim was to find a common vision of what would be effective, whether or not it was implementable at the current time, in view of the fact that Malaysia is a rapidly developing country and has very different levels of resource availability across the country [18]. A clear view of a desirable future is helpful in creating momentum and allowing people to work together.

## Methods

### Framing the research question and creating the first set of recommendations

The research question had originally come from a qualitative research project, which explored collaboration in the mental health system in Sabah, Malaysia. The first set of recommendations were produced by a local level committee in a psychiatric hospital, who met face to face, in the state of Sabah on the island of Borneo. This committee consisted of mental health staff and service users and had a total of eight meetings to produce the first set of recommendations. The committee were asked to create a set of recommendations for the hospital, based on qualitative research that had been conducted looking at collaborative practice in the hospital [8, 19]. Information from searches of research databases was also regularly presented at these meetings, where research questions arose from the discussion, focusing mainly on systematic reviews and meta-analyses (searches included the Cochrane Library, Embase, MEDLINE, Web of Science, Scopus, Psychinfo and Google Scholar). All these meetings were recorded and all but one of them was transcribed and coded, using the coding template derived from the original grounded theory study. The meeting minutes and items for the recommendations were created from this thematic analysis and then discussed at the next meeting. Some of the recommendations were implemented in the hospital and the results discussed at the meetings. Further discussion of the functioning of this committee and implementation of the recommendations will be discussed in separate papers.

#### Formation of the panel

People with a special interest in mental health systems and collaborative practice in Malaysia were identified. Most of the people suggested for the Delphi panel had been originally suggested by the hospital level committee, mentioned above, but others were found through reviewing the literature and by referral of other people recruited to the panel. Patient and carer representatives were identified through a snowballing mechanism, whereby opinion leaders were asked if there were any patients or carers who were active in support groups or in advocacy. Invitations to participate in the Delphi panel were sent to a total of 36 people, using a mixture of What’s App messaging, emails, phone calls and face to face discussions. The people invited included psychiatrists, psychologists, counsellors, psychiatric nurses, primary care doctors, public health professionals, NGO representatives and service users.

### Determination of the expert panel size

It was aimed to get approximately twenty members of the panel, in order to ensure that all members could be communicated with individually if needed. A panel size of approximately twenty has reasonable stability and scores have been shown to correlate well with larger panel sizes [13].

#### Creating the questionnaire

An initial questionnaire was developed using Google forms about the model of collaborative care developed in Sabah. A five-point Likert scale was used to assess each of the items. This questionnaire was piloted by sending to two members of the research team (SF, DF) who were not involved in the development of the questionnaire and were not from a mental health background and a psychiatrist who was not involved in the study or in the panel.

### Information provided to panel members to aid their judgements

The items were proceeded by an introduction to the concept of collaborative practice and a brief explanation of the results of the qualitative research. Most of the items were accompanied with a brief explanation or a link to other materials. All of the additional material had been agreed upon by the hospital level committee. The accompanying material was designed to be informative without creating a large reading burden for the members of the Delphi panel.

### Administering the questionnaire

Three Delphi rounds were conducted February 2018, July 2018 and February 2019. The questionnaire was sent to the panel of experts, providing an anonymous on-line mechanism for them to review and comment on the collaborative care model. Panel members were given approximately 1 month to complete each round, with several reminders sent during this period. Panel members were not reimbursed for their time. Panel members who completed round one, but did not complete round 2, were given the opportunity to take part in round 3, after informing them about previous results.

### Analysing rounds and providing feedback to the panel

An a priori decision was made that items were considered to have reached consensus if no members of the panel disagreed with an item and if the interquartile deviation was less than one [20]. The items were changed or removed if consensus had not been reached and the panel asked to re-rate the items and comment on them in the next round. The panel were sent anonymised ratings and comments from the previous round, which were displayed before asking the panel members to rate the item again. Where consensus had been reached, but comments were made suggesting minor changes to wording, the changes were made, and the panel was asked to comment. The panel was also asked to suggest any additional items during the first round of the Delphi process, and these were rated during the second round. The original hospital level committee discussed changes to the items before sending out for the final round as a way of reducing biases. The Delphi panel were also asked to rate and make comments about the process and changes were made to the process of subsequent rounds based on these comments.

### Reporting results

Written comments were imported into NVivo version 10. Initial open coding was conducted, followed by amalgamation of codes into higher level categories. WS conducted the initial coding and SF also examined the raw data and checked agreement with the codes. Comparison between patient/NGO comments and healthcare staff comments was conducted using a matrix coding query. The area of difference are highlighted in this paper in order to better understand how service user input into guideline development is important. Reporting was done using the CREDES statement for Delphi studies [21].

#### Ethics and consent

The study was approved by the Medical Research and Ethics Committee, Ministry of Health Malaysia (NMRR-13-308-14792). All hospital level committee members signed written informed consent forms to agree for the recording of the meetings to be used for the purpose of research. All Delphi panel members agreed to participate electronically, after personalised contact (through emails, messages and sometimes phone calls) to explain the process.

## Results

This paper will focus mainly on the Delphi panel, the functioning and effectiveness of the hospital level committee will be discussed in a separate paper.

### Composition of the hospital level committee and the Delphi panel

Table 1 shows the composition of the hospital level committee and the Delphi panel. There were 33 people who had attended the meetings of the hospital level committee, who were all based in one psychiatric hospital in Sabah. Twenty-two people agreed to take part in the Delphi panel, from different parts of Malaysia, and different institutions, including hospitals, universities and government institutes. Ten people did not reply, one declined because they felt it was not related to them and one suggested another person. Eighteen completed round one, 11 completed round two and 14 completed round three (three participants who did not complete round two subsequently completed round three). Four more patients were recruited during the first round, suggested by one of the Delphi panel members, who felt there were inadequate numbers of patients in the panel and was a member of a social media-based support group.

**Table 1:** Composition of the hospital committee and Delphi panel (participants that completed at least one round)

	Hospital level committee	Delphi panel
Academic psychiatrists	1	1
Assistant medical officer	7	
Carer	2	
Carer and psychoeducation officer		1
Child and adolescent psychiatrist	1	1
Clinical Psychologist	1	1
Community and liaison psychiatrist		1
Community and rehabilitation psychiatrist		1
Community psychiatrist		1
Counsellor	1	
Dietician	1	
Forensic psychiatrist	1	
General psychiatrist	2	3
Healthcare assistant	2	
Liaison psychiatrist		1
Medical Anthropologist		1
Mental health NGO		1
Mental health NGO and patient		1
Medical officer	4	
Nurse	7	
Occupational therapist	1	
Patient	1	4
Public health specialist	1	
Total	33	18

### Quantitative analysis of items by the Delphi panel

**Fig. 1:**
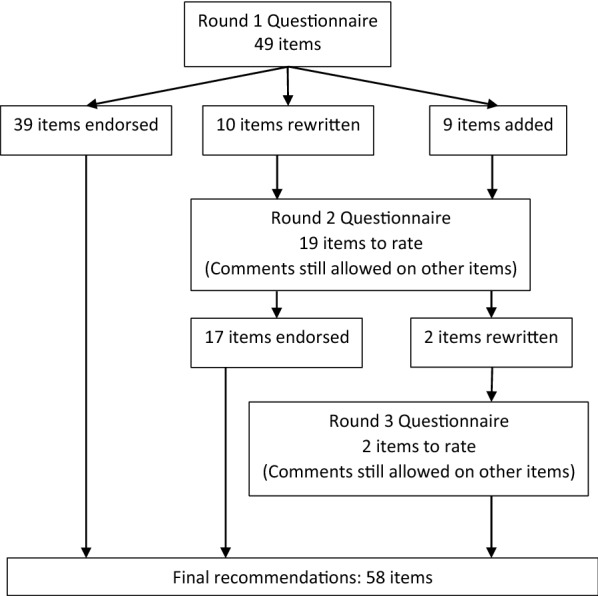
Flow diagram showing number of statements in each round

Table 2 shows a list of items, together with the mean response for each round and the interquartile deviation (IQD). Where items had reached consensus in the first round, there are no round 2 results shown. The items were categorised under themes: autonomy, relatedness, resources, collaborating outside the hospital and decision making. At the end of round one, 39 items had been endorsed and ten rejected. The ten rejected items were rewritten and nine new items were added before round two (see Fig. 1). All items except for two had reached consensus after two rounds. The two items which had not yet reached consensus were rewritten and then a third round conducted, with only these two items to rate. One of these items was the title. For this item, the respondents were given a choice of three titles and asked to give the preferred title and asked if they were acceptable. The chosen title was the preferred title and was considered acceptable by all participants. The full version of the recommendations can be found in Additional file 1. A full account of how the committee changed the items is in Additional file 2.

**Table 2:** Means and interquartile deviations of items

	Item	Round 1		Round 2	
		Mean	IQD	Mean	IQD
Title	Working Together: A Consensus on Collaborative Practice in the Malaysian Mental Health System	4.4	0.5	4.2	0.5
*Autonomy*					
1.1	The suggested process of collaborative problem solving and decision making should be considered as a way of empowering patients, carers and staff and improving the quality of decision making (as described in section 6 on previous page)	3.8	1	4.7	0.5
		*3 disagree*			
1.2	All staff should be trained in assertiveness, validation*, empathy and giving feedback* appropriately	4.7	0	4.8	0
1.3	Staff need to pay careful attention to furniture and subtle cues that may make people feel intimidated. In meetings we suggest that the seating should be as close as possible to circular, with no back row, if space allows	4.4	0.6		
1.4	The chair of the meeting should play a facilitator role and take care not to dominate	4.4	0.5	4.7	0.5
		*1 disagree*			
1.5	The chair of the meeting should be someone who has good meeting skills and skills in listening and validating, understands the topic and the context of the meeting and should be chosen with the agreement of the other members of the meeting. The chair should not be chosen purely on the basis of grade and profession			4.4	0.5
1.6	The meeting chair needs to create a non-judgmental, validating environment	4.3	0.5	4.8	0
		*1 disagree*			
1.7	The meeting chair needs to pay careful attention to power imbalances and make a special effort to elicit and validate opinions from people that may be feeling intimidated	4.5	0.5	4.6	0.5
		*1 disagree*			
1.8	Providing paper to people who might normally feel intimidated can encourage them to express themselves	4.3	0.5	4.3	0.5
		*1 disagree*			
1.9	Breaking up into smaller groups in larger meetings helps more voices to be heard and allows people to speak that normally feel intimidated	4.4	0.5	4.4	0.5
		*1 disagree*			
1.1	Staff in leadership roles should be mentored and trained in democratic and transformational leadership styles	4.6	0.5		
1.11	All staff should be given some leadership opportunities appropriate to their skills and experience. Junior staff should be given opportunities to chair meetings and mentored in this by more senior staff			4.7	0.5
1.12	The people involved in a meeting should be asked if they have any questions or feedback at the end of a meeting			4.8	0
Relatedness					
2.1	Systems should be designed so that there are as few transitions between healthcare providers as possible. If possible patients should see the same doctor on each visit	4.7	0.1		
2.2	A “primary nurse” system should be used for inpatients (see Additional file 2: Appendix S1)	4.8	0		
2.3	Systems should be designed in ways that optimize relatedness between staff	4.9	0		
2.4	Representatives (people that represent longer term committee members) should only be sent to patient care planning meetings or other hospital meetings when they are aware of the issues or are planning to join a hospital committee in the long term	4.4	0.5		
Resources					
Resources: staff competence and education					
3.1	All staff should be trained in the following areas: [insert list				
a	Interprofessional working	4.6	0.5		
b	Meeting skills	4.6	0.5		
c	Assertiveness skills	4.8	0.1		
d	Validating other people’s opinions and giving feedback	4.8	0.1		
e	Reflective practice	4.6	0.5		
f	Collaborative decision making and problem solving	4.6	0.5		
3.2	Training in collaborative competencies should be skills based and include role playing sessions and reflective components	4.5	0.5		
3.3	Most nursing and other professional staff working in psychiatric institutions should be interested in working in psychiatry and either have post-basic training in psychiatry or be undergoing this training	4.8	0		
3.4	Staff should be mentored. Staff with post-basic psychiatry training can mentor staff that do not have post-basic training	4.9	0		
3.5	Specific staff should be allocated to work in psychiatry in district hospitals and primary care, to allow these staff to develop the required competencies	4.6	0.5		
3.6	Higher authorities in the health service should ensure that there is an appropriate skills mix, i.e. that there are adequate numbers of all professional groups, including clinical psychologists and social workers	4.6	0.5		
Resources: service user competence and education					
3.7	The use of the Ministry of Health’s “Patient’s Unvoiced Needs” program, is recommended	4.6	0.5		
3.8	Each patient should have a written care plan, which they can share with all people involved in their care	4.6	0.5		
3.9	Education and support groups should be set up for patients and carers, including groups led by patients and carers. Brochures and promotional materials about existing groups should be made available in clinics and wards to ensure that patients, carers and staff are aware of their existence	4.8	0		
3.10	Patients and carers who are able and willing to help others should be trained to work as peer support workers and educators			5	0
3.11	Peer support workers and educators should be paid an honorarium for the time spent doing the work			4.4	0.5
3.12	Education for both the public and professionals should involve patients and carers as educators			4.8	0
3.13	Written materials should be available in doctor’s rooms or waiting room for patient and carer education, which should also be available on line. Patients and carers should be invited to write some of these materials if they are interested in doing this	4.7	0.1		
3.14	Information displayed on the wall of the clinic should be related to mental health, particularly ways to improve mental health and wellbeing. Information displays need to be clear, positive and sensitive to what patients may find distressing	4.6	0.5		
3.15	Mental health education videos should be shown in the waiting area of the clinic as well as being available online. These videos should show positive, hopeful, non-stigmatising views of mental illness	4.6	0.5		
3.16	A resource room or area should be available near the waiting room, which contains educational materials (brochures, books, videos). This should be staffed by someone capable of giving education to patients and carers, e.g. a staff member or peer educator	4.3	0.5		
3.17	We recommend that patients be given a clinic book. This book can be used for the following: Individualised care plans, recovery goals, relapse plans, education, psychological work—e.g. CBT formulation, pages to write down things that they would like to discuss with the doctor	4.7	0.1		
Resources: time					
3.18	Staffing calculations and rotas should take account of the time needed for collaboration	4.4	0.5		
3.19	Psychiatric appointment time should be at least 30 min for a follow up appointment and 90 min for a new patient appointment			4.5	0.5
3.20	Care needs to be taken in deciding how to use multi-professional meeting time. Topics of discussion should be limited to the things that concern most of the people attending the meeting	4.4	0.5		
3.21	Each member of staff should participate in only a limited number of hospital committees and junior staff should sometimes be appointed as committee members	4.6	0.5		
Resources: infrastructure					
3.22	Better physical resources are likely to improve collaborative practice	3.9	0.5		
Collaborating with people outside the hospital					
4.1	The bureaucratic processes should encourage collaboration, rather than create barriers to collaboration	4.5	0.5		
4.2	A ‘Friends of the Hospital’ group should be set up, together with a directory of services outside the hospital	4.4	0.5		
4.3	Specific mental health staff should form relationships with other people outside the hospital that help our patients	4.5	0.5		
4.4	Existing collaborative networks between primary care and people in the community should be used to help plan care for our patients (see Additional file 2: Appendix S1)	4.7	0.0		
4.5	Patients who are not directly under the psychiatric hospital, should be given the option of being treated in primary care (rather than district hospitals)	4.3	0.5	4.9	0.0
		*2 disagree*			
4.6	First-responder training programs in mental health should be provided for other people that help our patients	4.6	0.5		
4.7	Other people that help our patients need to know referral pathways and who to call if they are uncertain about what to do	4.7	0.3		
The decision-making process					
5.1	Inviting to take part in problem solving and decision making	4.7	0.5		
5.2	Identifying stakeholders	4.7	0.4		
5.3	Defining the problem			4.8	0.0
5.4	Finding common goals and values			4.8	0.0
5.5	Sharing of knowledge, opinions and concerns	4.6	0.4		
5.6	Making the final plan	4.4	0.4	4.5	0.5
		*2 disagree*		*1 disagree*	
	Consensus reached round 3: Mean 4.6, IQD 0.5				
5.7	Implementing the decision and making clear that the decision can be reviewed	4.8	0.0		

There are no round two results, where consensus was reached in the first round

*IQD* interquartile deviation

### Analysis of comments from Delphi panel

Most of the comments that both service users and staff made were broadly supportive of the guideline statements. The differences between the ways that staff and service users commented is highlighted.

#### Autonomy

All the respondents agreed with the need to empower patients and staff. Some service users described feeling intimidated and the difficulties that patients sometimes had in expressing themselves with doctors. Three new items were added after round one after suggestions from the panel, about ways to choose a meeting chair, giving leadership opportunities to staff and ensuring there was time for questions at the end of meetings.

#### Relatedness and continuity of care

Seeing a regular doctor appeared to be particularly important to service users. Service users commented extensively about this, illustrating with stories of difficulty in the system due to problems in continuity of care.

*D8: I have experience seeing the same doctor from 2011 to 2014 and it really helps me a lot! Now I have to see different doctors at every visit, and I feel lazy to tell my stories again and again. The communication is just superficial, I tell the surface stories and the doctor gives surface suggestions. No chance to explore further. After all, why share so much if I may not see him again?*

*D18: As a patient, I felt truly disconnected from my treatment plan because it was handled by different doctors. After moving to a hospital that assigns patients to the same doctor throughout their treatment process, I began to feel a sense of connection. My doctor knows my story from the beginning, so I didn’t have to keep repeating the same story. Repeating my story to different doctors when I was unstable prevented me from seeking help because I had the idea that nobody cares.*

Staff also agreed that this was important, but some had concerns about whether it was feasible for patients to regularly see the same doctor. Having a ‘primary nurse’ on the ward (a nurse case manager who cares for a patient throughout the length of their stay) also appeared to be more important to service users than staff.

#### Resources

Staff regularly mentioned resources, particularly not having enough staff in the system, not having enough time and how lack of resources made it difficult to implement some of the collaborative practice interventions. Service users focussed on the quality of staff and the problems associated with having undertrained staff, some commenting on how bad experiences with staff could hamper recovery.

Service users emphasised how people that had used the system were a useful resource and several of them commented on how they wanted to contribute, for example in producing educational materials. Service users placed a high value on help from people who had gone through similar experiences and some commented that service users could understand better than hospital staff. Three additional items were added after round two, which were related to peer support workers.

#### Working with people outside the healthcare system

All comments agreed that there was a need for better collaboration with people outside the hospital. There was concern about working with *Bomoh* (traditional healers) from some panel members:

*D11: Ambiguity will arise when we work with Bomohs. Does this mean that we accept what they are doing? Does this mean that we from the scientific “community” agree with supernatural existence as what is practiced by Bomohs? Clear rules should be set before working with unregistered authorities. Because it can always backfire on us.*

Some described out that the contribution of people outside the health system needed to be valued more and this may require a change in attitude from some of the staff.

*D16: The doctors and hospital staff must be open to this idea and must realize that everyone has their own area of expertise and knowledge. Sometimes because doctors and those from the medical field feel or regard themselves as the ‘experts’ it may hinder from us achieving this goal as they might not want to listen or take in the opinion of others.*

#### Decision making

This was the area with the most initial disagreement, particularly about the involvement of other people in decision making. Some panel members were concerned that involving other people in decision making might cause problems in confidentiality, be impractical and delay decision making The wording was changed to make it clear that this should be done with the permission of the patient, was optional and the time spent should be proportional to the difficulty and implications of the decision being made. There were also some concerns about patients that may not be able to make decisions for themselves, so further clarification was given on this in Additional file 2: Appendix S1. Additional items were added about defining the problem and setting goals, following suggestions from the panel.

#### Ratings and comments on the delphi process

Table 3 shows the ratings of panel members, regarding the Delphi process. These ratings were done at the end of round one.

**Table 3:** Participants experience of the Delphi process

Round 1	Strongly agree	Agree	Neutral	Disagree	Strongly disagree
I understand the rationale for the Delphi process	8	7	0	0	0
Filing the form was easy	3	10	0	2	0
Filling the form took longer than I expected	1	2	6	2	4
The Delphi process is a useful way to make new guidelines	4	8	3	0	0
Round 2					
I understand the rationale for the Delphi process	6	1			
Filing the form was easy	2	5			
Filling the form took longer than I expected	2	3	1	1	
The Delphi process is a useful way to make new guidelines	4	3			

The comments on the Delphi Process were generally positive, with panel members glad to have been given the opportunity to take part.

*D6 (staff): It was a good opportunity to learn from other professionals as well as patients and caregivers.*

Some members described feeling confused by some of the items and that not enough context was given to them in round one. A further introduction with more context was written for round two.

Service users described feeling empowered by the process, felt that their voices were being heard and that they were contributing.

*D8: I feel I am involved in nation*-*building and we are all working towards a better Malaysia, better society and better standard of living. Process is long but it is unavoidable. It is good that you give us a reasonable timeframe to allow us to take part according to our pace.*

Some commented that certain professions were missing (e.g. family medicine specialist, social worker) and some members felt that the balance between service users and professionals was not enough:

*D16: Thank you so much. I do feel that this is a great way to get our voices heard. However, the mix is not balanced hence the answers will always lean to a medical model rather than a social model and will again fall back to what the mental health professionals feel, think and want and do not fully represent what the patients and carers fully need and want.*

## Discussion

This process has created a set of recommendations, which aim to improve the general environment of the psychiatric system so that collaboration is more likely. The recommendations include ways to empower and improve autonomy, improvements in continuity of care and ways to enhance and make the best use of scarce resources. This is particularly important in Malaysia, where care is often fragmented [9] with low continuity of care [8], decision making is often hierarchical rather than collaborative [8], resources are limited [18, 22] and the treatment gap is large [10]. It is possible to implement many of these recommendations with the existing levels of resources. Although some of these recommendations are not feasible in many areas of Malaysia with the current level of resources, this exercise allowed consensus to be reached on what was desirable. Many of these recommendations already have empirical evidence to support them, which is briefly reviewed below.

### Autonomy

Responses by Delphi panel members demonstrated how low levels of autonomy negatively affects patient care. A large meta-analysis of 184 studies based on self-determination theory showed patient autonomy to be associated with mental health and physical health outcomes. This effect is likely to be motivated by perceived competence, whereby patients that feel more in control of their lives are more likely to feel competent in the management of their health [23]. The effects of interventions that aim to improve autonomy have been found to be greater in marginalised and disempowered groups such as people with low income or education levels [24, 25].

### Relatedness

The responses from service users on the Delphi panel highlight how inadequate continuity of care (e.g. patients seeing a different doctor on each visit) has a negative effect on care. Continuity of care is well studied and has been shown to be related to improved health outcomes [26], improved satisfaction [26, 27], improved cost effectiveness [26], decreased hospitalizations [27], decreased emergency department visits [27], and increased probability of receiving preventive services [27], particularly in patients with chronic diseases. A study using the French National Health Insurance database to follow up 14515 people with mental disorders for 3 years found relational continuity of care was related to reduced risk of death in people with mental disorders [28]. A UK longitudinal study examining the relationship between continuity of care in 5552 individuals with severe mental illness over 11 years showed that people with lower continuity of care had worse outcomes, with a large effect size of 1.75 (*Cohen’s d*) for the relationship between continuity of care and patient outcome [29]. Relatedness among staff is also important and a UK study of over 7000 health staff in 400 different healthcare teams showed that working in well-functioning teams led to lower levels of staff stress, lower death rates and higher levels of innovation [30].

### Resources

The original qualitative research showed that the level of resources limited collaboration, including the mental health and collaborative competencies of staff and service users, time and physical resources [8]. Meta-analytic evidence shows that interventions that improve the competence of healthcare staff, including educational meetings [31] and training in patient centeredness [32], improve patient outcomes. These interventions are more effective if the training involves mixed didactic and interactive elements [31]. Training of mental healthcare staff also reduces burnout [33] and the use of restraint [34]. Empathy training is effective in improving empathic responding in healthcare staff [35].

Psychoeducation programs that improve patient understanding of their illness improves patient outcomes, including compliance, relapse and satisfaction with services [36]. Individualised care planning improves the ability of patients with chronic illnesses to manage their condition as well as reducing depression [37] and qualitative evidence suggests that many patients value and use written care plans [38]. Discharge planning processes reduce the length of hospital stay, readmissions and improves patient satisfaction [39]. Handheld records have been shown to improve communication and patient knowledge in other disciplines [40, 41]. There is evidence that peer support interventions can improve patient outcomes, including reducing inpatient service use [42]. Research in Malaysia has shown that approximately 20% of patients have unvoiced needs following a doctor’s appointment [43] and a waiting room intervention led to reductions in unvoiced needs [44]. Waiting room interventions that help patients to identify their informational needs improve aspects of the consultation, including asking questions, patient satisfaction and pre-consultation anxiety [45]. Waiting room poster displays and educational brochures are read by patients in other contexts [46, 47], but there is currently little research into how these interventions affect health outcomes.

In the Malaysian setting a typical outpatient appointment lasts approximately 5–10 min and committee and panel members discussed how this resulted in patients feeling rushed. There is evidence in primary care that longer appointment times improve the detection of psychiatric problems [48]. There is little research into the optimum appointment length in psychiatry. In the US setting, appointments with psychiatrists are often reduced to a 15 min ‘medication check’, with the expectation that the patient will be seeing another professional for psychological interventions. This has led to dissatisfaction from both psychiatrists and patients and concerns that care is substandard [49]. In the Malaysian setting, patients are normally not seeing any other professional for psychological treatment and most patients are seen by inexperienced medical officers, rather than psychiatrists, so longer times are likely to be needed to provide adequate care.

Participants in this research commented on the way that physical infrastructure affected collaboration, for example the institutional feel of the wards reducing the sense of autonomy. Building design influences the way that people interact with each other [50–52] and architecture has the potential to increase or reduce the sense of power imbalance [53]. In a psychiatric setting, meeting spaces need to feel private and psychologically safe for patients and staff [53]. Hospital information systems can also improve collaboration in healthcare settings [54], improve communication between healthcare providers and service users [55] and improve accessibility of healthcare, leading to improved patient outcomes [56–59].

### Collaboration with people outside the hospital

Communities play a large role in mental health care in lower and middle income countries [60, 61] and partnership with communities is a strategy that has been successfully employed in Malaysia to improve mental health [62]. Training community members to provide initial help to people with mental disorders helps improve confidence, intention to help others and helping behaviours, however it is not yet clear whether these programs help improve mental health outcomes in people with mental health problems [63–65]. A systematic review of religious interventions concluded that they were effective [66] and in Malaysia religious professionals and traditional healers sometimes refer patients to services if they feel that the problem is a mental health concern, rather than a spiritual issue [19]. Interagency collaboration is considered best practice in the field of mental health, but the evidence that it is effective in improving patient outcomes was considered weak by a Cochrane review [66]. This is likely to be due to the complexity of these kind of interventions, where conducting a randomised control trial is difficult. However, a systematic review has shown that interagency collaboration has been shown to lead to better child welfare outcomes where there is parental drug use [66]. There is strong evidence that collaborations between primary care and specialist mental health staff are effective in treating people with mental disorders [7]. Treating patients with common mental disorders in primary care rather than secondary care has been recommended by the World Health Organisation for many years, since primary care is more accessible and acceptable to patients [61].

### The decision-making process

The process for shared decision making and problem solving that we have recommended has similarities and differences with processes for doctor-patient relationships previously described in the literature [67–73]. The step of ‘identifying stakeholders’ is unusual in models of doctor-patient decision making, since most models only concern the doctor and patient. In Malaysia decisions are frequently made outside of the doctor-patient dyad, with family and other community members often involved in decision making, even after the patient has left the doctors office [19]. Programs that aim to improve shared decision making have been shown to improve patient satisfaction and collaboration with the treatment process, but most studies do not show improvements in symptoms or behavioural outcomes [24, 74, 75].

### Complex multicomponent interventions

It appears that complex programs, which involve several of the components of collaborative practice (e.g. programs that increase patient education, autonomy and relatedness together), have an effect on more outcomes than programs which only introduce one component (e.g. only training in shared decision making) [7, 24, 76]. It is possible that the components of collaborative practice work synergistically, where several elements working together have a greater effect.

### Limitations

There are limitations to the way that we carried out this study. The first was that some groups were only represented in the original hospital committee, but not in the Delphi panel, particularly nursing and allied health staff. Service users were also underrepresented, particularly in the original hospital level committee and the service users on the committee are unlikely to have represented the views of all service users. Secondly, there was quite a large drop-out rate from the professionals in the Delphi panel. This is likely to reflect the reality of working in mental health in Malaysia, where it is extremely difficult to fulfil core work duties within working time and there is rarely time to do anything outside of core duties. This may have improved if panel members had been paid an honorarium for their time.

### Strengths

This study has shown that the Delphi method is a feasible method of making recommendations in mental health in Malaysia. A search of the literature did not reveal any other studies using this method in mental health in Malaysia, other than one pan-Asian study [77]. This method used minimal budget and has led to a more diverse group of people being involved in forming recommendations than is the case with traditionally used methods of decision making, which often only involve people in positions of power, who live in a small geographical area. This study attempted to give voice to those who have traditionally been left out of decision making. The World Health Organisation recommended in 2001 that “Communities, families and consumers should be included in the development and decision-making of policies, programmes and services” [78] and formal collaborations with service users is one of the WHO quality indicators [79]. This is not currently common practice in Malaysia [80] and is not one of the quality indicators commonly used [81]. This study has demonstrated the usefulness of involving consumers in forming recommendations, in that the perspective that they gave, and their priorities were different from the priorities of people that worked in the system. This was particularly the case with continuity of care, which appeared to be high priority for service users. This study demonstrated some of the difficulties of recruiting service users to committees in a setting where patriarchal attitudes to patients are prevalent [82], service user involvement is not common practice and there are still very few consumer groups. However, the process highlighted the existence of informal social media-based groups of mental health service users that are now growing and empowering users, one of which was eventually used to help recruit the patients and carers to the Delphi panel.

### Future directions

It is hoped that over time some of these recommendations will be implemented and incorporated into quality indicators of the Malaysian healthcare system. Further research is now needed into the effectiveness of some of these recommendations, in the context of Malaysia. Systems based research in lower and middle income countries is currently lacking, but was rated as high priority in study of researchers and stakeholders in low and middle income countries [83]. Research in lower and middle income countries is needed, particularly into the effectiveness of patient and staff empowerment, shared decision making, improving relatedness in the system, written care plans and information, increasing the provision of certain types of training and collaborating with the wider system.

## Conclusions

This study sought to build evidence on interventions which will help to improve patient care through improving collaborative practice in Malaysia. This has shown that the modified Delphi method is a feasible method in Malaysia and led to participation of a more diverse group of people than traditional methods of decision making. It also demonstrated the importance of involving service users and the challenges in doing this when it is not yet part of the culture. These recommendations could potentially be part of level III evidence [84], in the formation of clinical practice guidelines for complex systems level interventions, where higher level evidence is currently weak in Malaysia.

## Supplementary information

**Additional file 1.** The full guidelines.

**Additional file 2.** Detail about how items were modified and Delphi panel comments for each round.

## Data Availability

The anonymised, raw data from the Delphi Committee and the changes made to each item are available in Additional files 1, 2.
