# Supplementary material for: Creation of consensus recommendations for collaborative practice in the Malaysian psychiatric system: a modified Delphi study
Source: Int J Ment Health Syst. 2020 Jun 19;14:45. doi: 10.1186/s13033-020-00374-7 (PMC7304147; doi:10.1186/s13033-020-00374-7)
Supplement: Supplementary file 1 — Additional file 1. The full guidelines. [file 13033_2020_374_MOESM1_ESM.docx]

Working Together: A Consensus on Collaborative Practice in the Malaysian Mental Health System

Authorship team

Collaborative Practice Committee of Hospital Mesra Bukit Padang:

| Adam Abdullah | Assistant medical officer |
| --- | --- |
| Ahmad Qabil Bin Khalib | Director of Hospital Mesra Bukit Padang |
| Atiqah Chew Abdullah | Research |
| Brenagempli anak Jampong | Assistant medical officer |
| Cassie Ellena Chew | Nurse |
| Chua Sze Huang | Specialist |
| Cornelius John | Health care assistant |
| Ebba Gondilou | Nurse |
| Emmanuel Joseph Pereira | Specialist |
| Geo Allen George | Dietician |
| Julius Jalani | Research |
| Ms K | Carer |
| Kulnah Ambor | Nurse -Head of ward |
| Lee Sang Choon | Matron |
| Lee Shea Wah | Medical officer |
| Mary Malanjun | Health care assistant |
| Masnah Ajis | Nurse -Head of quality unit |
| Matron Nurzelah Abdullah | Matron |
| Ng Boon Seng | Medical officer |
| Norhayati Nordin | Hospital director (previous) |
| Nurmina Nawali | Carer |
| Ong Kok Loong @ Mohd Benyamin Abdullah | Patient |
| Razifah Adbul Rahman | Specialist |
| Rewajoh Lahai | Carer |
| Rommy B. Ramlee | Head OT |
| Segaran Ramodran | Associate Professor of Nursing |
| Shahlini Selvarajoo | Medical officer |
| Sharifah Aini Yusoff | Counsellor |
| Sidik Singkak | Assistant medical officer |
| Sughashini Subramaniam | Medical officer |
| Wendy Shoesmith | Research |
| Zaidin Endut | Head medical assistant |

Delphi Committee:

| Abdul Kadir Abu Bakar | Community and Liaison Psychiatrist |
| --- | --- |
| Abdul Rasyid Sulaiman | Psychiatrist at International Medical Univerisity |
| Ahmad Rostam Md Zin | President of the Early Career Psychiatrists section of Malaysian Psychiatric Association |
| Amalina Abdullah | Psychiatrist at Hospital Batu Pahat |
| Ang Kim Teng | Hon. Secretary General, Malaysian Mental Health Association |
| Anita Abu Bakar | President of Mental Illness Awareness and Support Association (MIASA). |
| Low Lee Lan | Medical Anthropologist at MOH Institute for Health Systems Research |
| Marhani Midin | Community psychiatrist with interest in psychotherapy |
| Ms A | Patient |
| Ms N | Patient |
| Nurul Nadia Ismail | Psychiatrist at Hospital Sultan Abdul Halim, Sungai Petani |
| Shazwani Rhosky binti Fadzir | Patient |
| Siti Hazrah Selamat Din | Community and Rehabilitation Psychiatrist |
| Tang Poh Yee | Psychoeducation officer and carer |
| Umi Izzatti Saedon | Clinical Psychologist |
| Wan Zakirah bt. Wan Zakaria | Patient |
| External Advisors | |
| Sue Fyfe |  |
| Dawn Forman |  |
| Beena Giridharan |  |
| Some members chose to remain anonymous or were not contactable at the time of publication | |

Introduction

Collaborative practice has been defined by the WHO as “when multiple health workers from different professional backgrounds work together with patients, families, carers and communities to deliver the highest quality of care”^1^.

Collaborative practice improves the quality of healthcare delivery. Collaboration can be between different professional groups, between patients and healthcare providers, between different parts of the healthcare system, or between the healthcare system and other people that help patients. The effect of collaboration is similar in size to the effect of many commonly used psychiatric medications.

Collaboration between different professions reduces admissions, length of admission and mortality, and improve satisfaction, employment and accommodation status^2–9^. Therapeutic alliance between mental health providers and patients is one of the best predictors of outcome^10–16^. Collaboration between families and professionals has been found to be the most important predictor of carer satisfaction with services ^17,18^. Collaboration between primary and secondary care has been shown to significantly improve outcomes in anxiety and depression^19^. Collaboration between health and social care agencies leads to improvements in symptoms and functioning in people with mental disorders^20^.

Collaborative practice interventions are often complex and not standardized. These kinds of interventions are difficult or very expensive to assess using clinical trials and results of trials cannot easily be generalized from one context to another. Consensus methods, such as the Delphi technique, are a way of providing guidance on complex systems level interventions in healthcare^21,22^.

These recommendations are a consensus of what is desirable in the system, whether or not it is possible to implement at the current time, in order to create a clear view or a desirable future.

Guideline Development

These guidelines were produced as part of an action research project, which started in 2013. The project had a number of phases, which are listed below:

Phase 1

Interviews and focus groups were held with staff, patients, carers and other people that help people with mental disorders (e.g. religious authorities, village leaders, school counsellors, NGO workers, traditional healers). A literature review was conducted.

Phase 2

A committee was formed consisting of staff from a psychiatric hospital, patients and carers. The group considered ways of improving collaborative care in the hospital. A consensus process was used to produce the guidelines. The model was then reviewed by a nationwide Delphi committee.

Phase 3.

Where possible the guidelines were implemented in a psychiatric hospital and the results reflected upon.

Barriers to collaborative practice in Malaysia

Collaboration has large benefits for both patients and staff, but it is not easy to achieve. Below are some of the barriers to collaboration described by staff in the Malaysian healthcare system^23^:

Hierarchical environment and lack of autonomy

Staff describe how collaboration is difficult in an environment where lower ranking staff are afraid of expressing their opinions to higher ranking staff and patients are afraid to express their opinions to doctors. Adequate autonomy is needed to engage in collaborative decision making. Malaysia has one of the most hierarchical cultures in the world and staff describe how this has an effect on patient care^24–26^.

Lack of relatedness

Trusting relationships are needed for good collaboration. If patients see different doctors on each visit and staff do not work with a familiar team then trusting relationships fail to develop.

Lack of resources

This includes time, competencies and physical resources.

Lack of motivation

Some staff reported that they are burned out or not always motivated to provide the best possible care for patients or to collaborate with other staff, patients and carers. Some described seeing this problem in others.

Producing a collaborative environment in Psychiatric Settings

We believe that the following ingredients are needed to create a collaborative environment.

Autonomy

Patients, carers and staff need to feel that they have the autonomy to be involved in decision making.

Relatedness

Relatedness is the basic psychological need to “form and maintain strong, stable interpersonal relationships”^27^. Patients, carers and staff need sustained, trusting relationships in order to collaborate.

Resources

Resources include competencies, time, physical resources and opportunities. Competencies in collaboration, psychiatry and mental health are needed in all grades of staff, patients and carers.

These ingredients are the basic psychological needs that create internal **motivation** in both staff and patients^28^. Collaboration requires all parties to be motivated to move towards **common goals and values**. These ingredients should increase motivation in all parties to create the best possible outcomes for patients and to collaborate.

We recommend that consideration is given to ways of improving each of these ingredients.

# Improving Autonomy

The following are suggested ways to increase autonomy and reduce the sense of hierarchy in meetings. (These may be large interprofessional meetings or smaller meetings between doctor and patient).

## The suggested process of collaborative problem solving and decision making should be considered as a way of empowering patients, carers and staff and improving the quality of decision making (see section 5).

## All staff should be trained in assertiveness, validation*, empathy and giving feedback* appropriately.

Staff should be trained to use these skills when communicating with other staff, as well as with patients and families.

###### *Validation means acknowledging and accepting the thoughts and feelings of others, without necessarily agreeing with them. Appropriate forms of feedback include the 'sandwich method' and the 'ALOBA' method.

## Staff need to pay careful attention to furniture and subtle cues that may make people feel intimidated.

In meetings we suggest that the seating should be as close as possible to circular, with no back row.

## The chair of the meeting should play a facilitator role and take care not to dominate.

## The chair of the meeting should be someone who has good meeting skills and skills in listening and validating, understands the topic and the context of the meeting and should be chosen with the agreement of the other members of the meeting. The chair should not be chosen purely on the basis of grade and profession.

## The meeting chair needs to create a non-judgmental, validating environment.

They need to ensure that people are not denigrated by other people in the meeting. This is particularly important for more junior staff.

## The meeting chair needs to pay careful attention to power imbalances and make a special effort to elicit and validate opinions from people that may be feeling intimidated.

## Providing paper to people who might normally feel intimidated can encourage them to express themselves.

This is more relevant to large staff meetings, where some staff may feel intimidated, even with assertiveness training. Brainstorming sessions, where staff are asked to write ideas and put them in a box can also save time in a large meeting.

## Breaking up into smaller groups in larger meetings helps more voices to be heard and allows people to speak that normally feel intimidated.

## Staff in leadership roles should be mentored and trained in democratic and transformational leadership styles.

These leadership styles both emphasize autonomy, engagement, values and internal motivation, rather than leading by using threats and rewards as a form of motivation.

## All staff should be given some leadership opportunities appropriate to their skills and experience. Junior staff should be given opportunities to chair meetings and mentored in this by more senior staff.

## The people involved in a meeting should be asked if they have any questions or feedback at the end of a meeting.

This includes asking patients if they have feedback at the end of a consultation.

# Improving Relatedness

The following are recommended to improve relatedness in the system, which will allow trusting relationships to develop.

## Systems should be designed so that there are as few transitions between healthcare providers as possible. If possible patients should see the same doctor on each visit.

Patients report that they do not like having to tell the same story many times to different doctors and then never seeing them again. If they are unable to form a relationship with their healthcare provider then a therapeutic alliance will not form. Healthcare providers also report that they are more satisfied if they follow up their own patients and most report it is more efficient than seeing patients they have never met. This is particularly important in the early stages of treatment, where illness is unstable and where patients have not yet returned to previous levels of functioning. This may need to be balanced against the training needs of junior staff and service flexibility. If patients are not recovering then they should be discussed in supervision or reviewed by another doctor.

## A “primary nurse” system should be used for inpatients.

This allows inpatients to have one nurse that they are familiar with throughout their stay. This nurse will be responsible for the medium- long term care needs of the patient, including patient education, discharge planning, liaising with other professionals and forming a relationship with the patient’s family. This nurse will not be on every shift, but may see the patient several times per week. We recommend that an “associate nurse” is also appointed for each patient to cover the primary nurse. Nurses need to be given training on how to fulfill this role.

## Systems should be designed in ways that optimize relatedness between staff.

Dividing the staff into multiprofessional teams is one way of increasing relatedness between staff and has been shown to significantly improve outcomes (23,24). This is already being done in the creation of community mental health centers. Teams could care for a particular group of patients (e.g. drug users, adolescents) or they could care for a particular geographical area. These teams could include staff members covering different parts of the hospital i.e. staff from wards, clinics and the community would be on the same team. Training needs could be met by rotating staff within the same team, without disrupting relationships.

The number of other staff that each person needs to collaborate with needs to be manageable.

## Representatives (people that represent longer term committee members) should only be sent to patient care planning meetings or other hospital meetings when they are aware of the issues or are planning to join a hospital committee in the long term.

# Improving Resources

Competence

Developing collaborative competence of staff

Collaborative competencies are complex and generally do not develop unless staff are specifically trained in them.

## All staff should be trained in the following areas:

- Interprofessional working
- Meeting skills
- Assertiveness skills
- Validating other people’s opinions and giving feedback
- Reflective practice
- Collaborative decision making and problem solving (see section 6)

## Training in collaborative competencies should be skills based and include role playing sessions and reflective components.

Training is based around the principles of interprofessional education where staff learn from, with and about one another’s roles.

Developing mental health competencies in staff

## Most nursing and other professional staff working in psychiatric institutions should be interested in working in psychiatry and either have post-basic training in psychiatry or be undergoing this training.

Recognition needs to be given that psychiatric nursing is a specialized field and requires the application of multiple higher level skills.

## Staff should be mentored. Staff with post-basic psychiatry training can mentor staff that do not have post-basic training.

Staff working in psychiatric environments need regular supervision meetings, which are reflective and supportive.

## Specific staff should be allocated to work in psychiatry in district hospitals and primary care, to allow these staff to develop the required competencies.

These staff can be mentored by staff with post-basic psychiatric training.

## Higher authorities in the health service should ensure that there is an appropriate skills mix, i.e. that there are adequate numbers of all professional groups, including clinical psychologists and social workers.

There is a particular shortage of some groups of allied health staff, which makes interprofessional collaborative practice difficult.

Developing collaborative competencies in patients and carers

## The use of the Ministry of Health's “Patient’s Unvoiced Needs” program, is recommended.

A video is shown in the waiting room, which encourages patients to write down what they would like to discuss with the doctor before the appointment. This was introduced because it was discovered that many patients do not discuss what they intended to discuss with their doctor.

Developing mental health competencies in patients and carers

## Each patient should have a written care plan, which they can share with all people involved in their care.

This would be produced at care planning meetings attended by the primary nurse, primary doctor, patient, family and all others involved in their care.

###### A care plan is a list of actions which will help the patient move towards recovery goals and staying well. This can include actions provided by healthcare professionals (e.g. prescribing medication), actions done by the patient themselves (e.g. exercise) and actions done by family members (e.g. listening if the patient wants to talk). Writing the plan is a way of making sure that all members of the team, including the patient and family, know what the plan is and are working together towards the same goals. The complexity of the plan depends on the needs of the patients and a simple plan may just consist of a handwritten list of recovery goals and actions written in the patients clinic notebook.

## Education and support groups should be set up for patients and carers, including groups led by patients and carers. Brochures and promotional materials about existing groups should be made available in clinics and wards to ensure that patients, carers and

## Patients and carers who are able and willing to help others should be trained to work as peer support workers and educators.

## Peer support workers and educators should be paid an honorarium for the time spent doing the work.

## Education for both the public and professionals should involve patients and carers as educators.

## Written materials should be available in doctor’s rooms or waiting room for patient and carer education, which should also be available on line. Patients and carers should be invited to write some of these materials if they are interested in doing this.

## Information displayed on the wall of the clinic should be related to mental health, particularly ways to improve mental health and well being. Information displays need to be clear, positive and sensitive to what patients may find distressing.

## Mental health education videos should be shown in the waiting area of the clinic as well as being available online. These videos should show positive, hopeful, non-stigmatising views of mental illness.

## A resource room or area should be available near the waiting room, which contains educational materials (brochures, books, videos). This should be staffed by someone capable of giving education to patients and carers, e.g. a staff member or peer educator.

## We recommend that patients be given a clinic book. This book can be used for the following: Individualised care plans, recovery goals, relapse plans, education, psychological work – e.g. CBT formulation, pages to write down things that they would like to discuss with the doctor.

###### This may be in paper form or written form.

Time

Collaboration can initially take a lot of time. Time can sometimes be saved in the longer term, for example if it prevents a patient being readmitted. The following are suggested to ensure that collaboration time is used appropriately.

## Staffing calculations and rotas should take account of the time needed for collaboration.

Time needed for collaboration includes time for phone calls to other healthcare staff or families, time for specialists to provide consultation to medical officers, as well as time needed for meetings.

## Psychiatric appointment time should be at least 30 minutes for a follow up appointment and 90 minutes for a new patient appointment.

This includes the time needed for documentation, consultation from the specialist and discussion of the plan.

## Care needs to be taken in deciding how to use multi-professional meeting time. Topics of discussion should be limited to the things that concern most of the people attending the meeting.

If staff are unable to contribute at the meeting, or learn from it, then their need to attend should be reviewed.

## Each member of staff should participate in only a limited number of hospital committees and junior staff should sometimes be appointed as committee members.

This will reduce the load on more senior staff and empower more junior staff.

Physical Resources

## Better physical resources are likely to improve collaborative practice

– e.g. adequate inpatient infrastructure, interview rooms, rooms for group sessions, meeting rooms, IT facilities.

# Collaborating with Other People that Help People with Mental Disorders

Patients with psychiatric disorders are helped by many people outside of the formal psychiatric system, including school and religious counsellors, primary healthcare clinics, religious authorities, Bomohs and employers. Collaborating with these groups can increase the resources available to help patients. Collaborating with these groups will also be improved with adequate autonomy, relatedness and competence.

Autonomy

## The bureaucratic processes should encourage collaboration, rather than create barriers to collaboration.

Bureaucratic barriers to collaboration include requirements for staff to fill a form on leaving the hospital. The autonomy given to staff and teams needs to be determined individually, depending on skills, job requirements and experience.

Relatedness

## A ‘Friends of the Hospital’ group should be set up, together with a directory of services outside the hospital.

This will help form relationships with people outside the hospital to donate resources or services and lobby to improve resources available.

## Specific mental health staff should form relationships with other people outside the hospital that help people with mental disorders.

e.g. a specific specialist or other member of staff is responsible for providing training and support for a particular primary care clinic, school, or village.

## Existing collaborative networks between primary care and people in the community should be used to help plan care (see appendix).

## Patients who are not directly under the psychiatric hospital, should be given the option of being treated in primary care (rather than district hospitals).

Primary care clinics have existing collaborative networks with community members, such as village heads. It is also recommended by the World Health Organization and is more convenient for patients.

Resources

Improving community mental health competence

## First-responder training programs in mental health should be provided for other people that help people with mental disorders.

e.g. teachers, religious leaders, human resource staff

## Other people that help people with mental disorders need to know referral pathways and who to call if they are uncertain about what to do.

# Process of Shared Decision Making

COLLABORATIVE PROBLEM SOLVING AND DECISION MAKING

The following is a description of steps for optimal problem solving and decision making. These steps can be used in consultations with patients, care planning meetings and other hospital meetings.

These steps were written after qualitative research showed:

*• Patients, carers and some staff felt that they were not invited to take part in decision making, they were not asked their opinion, or that their opinion was not listened to. They described being asked for information but not being asked for their opinions about what was causing the problem or what should be done.*

*• Many participants (patients, carers and staff) described how they often felt that bad decisions were being made, but did not feel they could question the decision. They also sometimes felt that they could not tell doctors when a plan was not effective.*

*• Decisions were often made outside of the doctor-patient consultation or meeting. An example is where the patient decides to take medication during the consultation with their doctor, but then changes their mind after talking to a family member. In the process we describe below, the opinions of other people are deliberately discussed and considered while making shared decisions in a meeting.*

*• These problems were described in many different types of meeting, including patient-doctor consultations, ward rounds and general hospital meetings.*

The steps written are general guidance and the amount of time put into this process will depend on the problem to be solved or the decision being made. Not all steps need to be followed each time.

## Inviting to take part in problem solving and decision making.

Patients, carers and some staff may need to be explicitly asked if they would like to take part in the problem solving and decision-making process (see appendix below).

###### Patients, carers and some staff sometimes make the assumption that they are not expected to take part in decision making. An explicit invitation reduces this assumption. In patient consultations a simple invitational statement or question can be used: e.g. "It sounds like the sleepiness is causing you lot of problems. Shall we think together about what we can do about it?"

###### Some patients and carers may not wish to take part in decision making and problem solving, particularly if they are feeling overwhelmed or if the treatment options are complex. For example, sometimes patients and families prefer their doctor to select the medication that they feel is best for them. In these cases, the practitioner needs to decide whether it is therapeutically appropriate to a) respect their wish and make the decision for them or b) further encourage the patient to be involved in making the decision..

## Identifying stakeholders.

The people that are either affected by the problem or could help with the problem need to be identified (see appendix below). Decision making needs to be done at an optimal time, with these people present if possible. If they cannot be present at the time of decision making, then knowledge, opinions and concerns should be asked from them first. This should be done with the patient's agreement (see appendix).

###### People affected by the decision normally include the patient and family. People that may help with the problem include patient, family, healthcare staff and other people in the community, such as employers, teachers and community leaders. If healthcare staff have been previously involved and know the patient well, then their opinion should be asked before important decisions are made. Contacting other people must be done with the permission of the patient, including contacting family members. If the patient does not have capacity to make the decision about involving other people, then the decision to contact them should be made in the best interest of the patient, taking into consideration the balance of risk, the opinions of other people close to the patient, legal requirements and any advanced directives. The amount of time taken in taking opinions from different people needs to be proportionate to the importance of the decision, who else the decision will affect, the urgency in making the decision and the resources available. For example, more time will be spent asking different opinions about the decision to discharge a high risk patient than would be spent asking opinions about which medication to choose. In many cases it is appropriate to just ask the patient about the opinions of other people, eg "What does your family think about you taking medication?, "Are their people in your family who do not think it is a good idea?", "What do you think about your aunt's belief that taking medication will cause kidney problems?".

## Defining the problem.

The problem needs to be clearly defined and agreed upon.

###### In patient consultations the problem this may first involve exploring the patient's goals or values and working out the barriers to reaching these goals or following values.

## Finding common goals and values.

Common goals or values in solving the problem should be defined and agreed upon.

###### Goals involve a defined end point (e.g. finding a job), values give a general direction (e.g. would like to contribute). Goals should be found that all parties would like to work towards.

## Sharing of knowledge, opinions and concerns.

Knowledge, opinions and concerns, which will help with problem solving should be shared between the people present.

###### This process can involve a formal problem solving technique, starting with a brainstorming, where all possible ways of solving the problem are written down and the pros and cons of each solution discussed.

###### This process may need to involve people outside the meeting, in which case it may be necessary to defer decision making until all parties have shared information, opinions and concerns. For example, a patient may wish to defer a decision about switching medication until the next appointment, after sharing information about a new medication with family members and finding out their opinions.

## Making the final plan.

After deliberating the options, the plan should be produced. This should be written down for the patient if it is complex. The opinions of people not physically present or not capable of decision making at the time shoud be considered if they are relevant.

Changed from: All opinions should be taken into account when making the final decision, including opinions of people not physically present or not able to make decisions.

###### Patients may not have the capacity to make a decision at the point the decision has been made. However, many patients will have discussed what they want to be done in the event of future relapse. This may be in the form of a formal advance directive or relapse plan or they may have informally discussed this with a relative or member of the healthcare team. An example of this is a patient who writes a relapse plan which requests ECT in the event of becoming manic. This request would be considered by the healthcare team, when making decisions about ECT.

## Implementing the decision and making clear that the decision can be reviewed.

Discussion should take place about who should do what. A review date should be set. It should be made clear to patients and carers that the decision can be reviewed if the solution is not effective or makes the problem worse.

# References

1. World Health Organization. *Framework for Action on Interprofessional Education & Collaborative Practice*. Geneva: Department of Human Resources for Health; 2010. doi:10.1111/j.1741-1130.2007.00144.x

2. O’Mahony S, Mazur E, Charney P, et al. Use of multidisciplinary rounds to simultaneously improve quality outcomes, enhance resident education, and shorten length of stay. *J Gen Intern Med*. 2007;22(8):1073-1079. doi:10.1007/s11606-007-0225-1

3. World Health Organisation. *Framework for Action on Interprofessional Education and Collaborative Practice*.; 2011. http://espace.library.uq.edu.au/view/UQ:233239. Accessed May 9, 2012.

4. Holland R, Battersby J, Harvey I, Lenaghan E, Smith J, Hay L. Systematic review of multidisciplinary interventions in heart failure. *Heart*. 2005;91(7):899-906. doi:10.1136/hrt.2004.048389

5. Zwarenstein M, Goldman J, Reeves S. Interprofessional collaboration : effects of practice-based interventions on professional practice and healthcare outcomes ( Review ). *Cochrane Database Syst Rev*. 2009;(3):CD000072. doi:10.1002/14651858.CD000072.pub2

6. Malone D, Marriot S, Newton-Howes G, et al. Community mental health teams (CMHTs) for people with severe mental illnesses and disordered personality (review). *Cochrane database Syst Rev*. 2007;(3). doi:10.1002/14651858.CD000270.pub2.

7. Simmonds S, Coid J, Joseph P, Yrer PT. Community mental health team management in severe mental illness : a systematic review. *Br J psychiatry*. 2001;178:497-502.

8. Marshall M, Lockwood A. Assertive community treatment for people with severe mental disorders ( Review ). *Cochrane database Syst Rev*. 2005;(2).

9. Botha U a, Koen L, Joska J a, Hering LM, Oosthuizen PP. Assessing the efficacy of a modified assertive community-based treatment programme in a developing country. *BMC Psychiatry*. 2010;10:73. doi:10.1186/1471-244X-10-73

10. Horvath AO, Symonds BD. Relation between working alliance and outcome in psychotherapy: A meta-analysis. *J Couns Psychol*. 1991;38(2):139-149. doi:10.1037/0022-0167.38.2.139

11. Martin DJ, Garske JP, Davis MK. Relation of the therapeutic alliance with outcome and other variables: a meta-analytic review. *J Consult Clin Psychol*. 2000;68(3):438-450. doi:10.1037/0022-006X.68.3.438

12. McCabe R, Bullenkamp J, Hansson L, et al. The therapeutic relationship and adherence to antipsychotic medication in schizophrenia. *PLoS One*. 2012;7(4). doi:10.1371/journal.pone.0036080

13. Hopkins M, Ramsundar N. Which factors predict case management services and how do these services relate to client outcomes? *Psychiatr Rehabil J*. 2006;29(3):219-222. doi:10.2975/29.2006.219.222

14. Neale MS, Rosenheck RA. Therapeutic alliance and outcome in a VA intensive case management program. *Psychiatr Serv*. 1995;46(7):719-721. doi:10.1176/ps.46.7.719

15. Solomon P, Draine J, Delaney MA. The working alliance and consumer case management. *J Ment Health Adm*. 1995;22(2):126-134. doi:10.1007/BF02518753

16. Svensson B, Hansson L. Therapeutic alliance in cognitive therapy for schizophrenic and other long-term mentally ill patients: development and relationship to outcome in an in-patient treatment programme. *Acta Psychiatr Scand*. 1999;99(4):281-287. doi:10.1111/j.1600-0447.1999.tb07226.x

17. Perreault M, Rousseau M, Provencher H, Roberts S, Milton D. Predictors of caregiver satisfaction with mental health services. *Community Ment Health J*. 2012;48(2):232-237. doi:10.1007/s10597-011-9403-z

18. DeChillo N. From paternalism to partnership: Family and professional collaboration in children’s mental health. *Am J Orthopsychiatry*. 1994;(64):564-574. http://onlinelibrary.wiley.com/doi/10.1037/h0079572/abstract. Accessed November 21, 2012.

19. Archer J, Bower P, Gilbody S, et al. Collaborative care for people with depression and anxiety. October 2012. http://summaries.cochrane.org/CD006525/collaborative-care-for-people-with-depression-and-anxiety. Accessed July 11, 2013.

20. Hayes SL, Mann MK, Morgan FM, Kelly MJ, Weightman AL. Collaboration between local health and local government agencies for health improvement. In: Mann MK, ed. *Cochrane Database of Systematic Reviews*. Chichester, UK: John Wiley & Sons, Ltd; 2012. doi:10.1002/14651858.CD007825.pub6

21. Jones J, Hunter D. Consensus methods for medical and health services research. *Br Med J*. 1995;311(August):376-380.

22. Minas H, Jorm AF. Where there is no evidence : use of expert consensus methods to fill the evidence gap in low-income countries and cultural minorities. *Int J Ment Health Syst*. 2010;4(1):33. doi:10.1186/1752-4458-4-33

23. Shoesmith W, Awang Borhanuddin AF Bin, Pereira EJ, et al. Barriers and enablers to collaboration in the mental health system in Sabah, Malaysia: towards a theory of collaboration. *BJPsych Open*. 2020;6(1):1-10. doi:10.1192/bjo.2019.92

24. Hofstede G. Intercultural Co-operation in Organisations. *Manag Decis*. 1982;20(5):53-67.

25. Kennedy J, Mansor N. Malaysian Culture and the Leadership of Organizations - A Globe Study. *Malaysian Manag Rev*. 2000;35(2).

26. Shoesmith W, Sawatan W, Abdullah AFB, Fyfe S. *Leadership and Evaluation Issues in Interprofessional Education in Sabah, Malaysia*.; 2016. doi:10.1057/978-1-137-53744-7_10

27. Baumeister RF, Leary MR. The need to belong: desire for interpersonal attachments as a fundamental human motivation. *Psychol Bull*. 1995;117(3):497-529. doi:10.1037/0033-2909.117.3.497

28. Ryan RM, Deci EL. Self-determination theory and the facilitation of intrinsic motivation, social development, and well-being. *Am Psychol*. 2000;55(1):68-78. http://www.ncbi.nlm.nih.gov/pubmed/11392867.
