# Supplementary material for: Creation of consensus recommendations for collaborative practice in the Malaysian psychiatric system: a modified Delphi study
Source: Int J Ment Health Syst. 2020 Jun 19;14:45. doi: 10.1186/s13033-020-00374-7 (PMC7304147; doi:10.1186/s13033-020-00374-7)
Supplement: Supplementary file 2 — Additional file 2. Detail about how items were modified and Delphi panel comments for each round. [file 13033_2020_374_MOESM2_ESM.docx]

|  | Round 1 item: **Comments on introduction**  *Service user/NGO comments: ***Clear and concise****  *Healthcare staff comments: Ok***I feel that with this new introduction, health care professionals will be reminded to empower patients and carers in the management of their care management. An invitation would mean that the health care professional is aware that patients' and carers' opinions matter.***Can we do it in a point form? it is lengthy and wordy***The second last sentence probably can be revised to 'the steps written are the general guidelines...'.- to emphasized that this is just a guideline which can be used as a point of reference but not to be strictly followed. I think last sentence can be deleted.***A little bit lengthy, but examples are straightforward and easy to understand ***yes agree**** |  |
| --- | --- | --- |
| Title | Round 1 item: **Consensus Guidelines for Collaborative Practice in Mental Health Services for Malaysia**  *Service user/ NGO comments: ***i agree on the title***Possibly a more catchy title to move away from sounding so academic****  *Healthcare staff comments: ***Suggestion: Guidelines for Collaborative Practice in Mental Health Services for Malaysia***drop the word 'guideline'***Acceptable***The word consensus carries a lot of importance here, showing that the idea is from a group of people, after several discussions. Usually the title will just start with Guidelines for....I believe this title is the most apt.***Clear and concise*  Round 2 item: **Working Together: Guidelines for Collaborative Practice in Mental Health Services for Malaysia**  *Service user/NGO comments: I favour new title more than previous one***Good title, easier to understand***How about dropping guideline- quite academic still thankyou****  *Healthcare staff comments: I believe the word guidelines should be dropped. Working together: consensus for collaborative practice***The addition of "Working together" makes it more attractive and emphasises the idea of collaborative practice.***more inviting and sounds more 'consensus'***Agree. Clear and concise***yes agree with the edited title****  Round 3 item: **Choice 1: Creating the Conditions for Collaboration in the Malaysian Mental Health System: a Consensus**  **Choice 2: Working Together: Creating the Conditions for Collaboration in the Malaysian Mental Health System (a consensus)**  **Choice 3: Working Together: A Consensus on Collaborative Practice in the Malaysian Mental Health System** | Mean round 2: 4.2  IQD round 2: 0.5  1 respondents disagree |
| 1.1 | Round 1 item: **The suggested process of collaborative problem solving and decision making should be followed if complex or important decisions are being made (as described in section 6 on previous page).**  *Service user/ NGO comments: Patients and caregivers' voice is often neglected.****  *Healthcare staff comments: ***...unless the team get into consensus that they need to be more creative in solving the problem by not sticking to the generic processes***Need to overcome reticence of many Malaysians to offer opinions***the term complex and important is very subjective. Who is to decide whether the decision making is complex or important? It would be nice if all cases are regarded as important.****  Round 2 item: **The suggested process of collaborative problem solving and decision making should be considered as a way of empowering patients, carers and staff and improving the quality of decision making (as described in section 6 on previous page).**  *Service user/NGO comments: ***Ok***This will in my opinion strengthen the quality of decision making****  *Healthcare staff comments: ***I agree that the modification makes the process sound non-obligatory, and serves as an option to be considered.**** | Mean round 1: 3.8  IQD round 1: 1.0  3 respondents disagree  Mean round 2: 4.7  IQD round 2: 0.5  Consensus reached |
| 1.2 | Round 1 item: **All staff should be trained in assertiveness, validation* and giving feedback* appropriately.**  **Validation means acknowledging and accepting the thoughts and feelings of others, without necessarily agreeing with them. Appropriate forms of feedback include the 'sandwich method' and the 'ALOBA' method.*  *Service user/ NGO comments: This will improve patient experience.***this is important skill needed for the staff***Good models of giving feedback***Indeed. This ensures that information relayed is correct and accurate.****  *Healthcare staff comments: ***and more importantly to LISTEN to the patient's complaint/problem***Agreed***Not only validation, but also empathy needs to be trained and incorporated into everyone who is involved.****  Round 2 item: **All staff should be trained in assertiveness, validation*, empathy and giving feedback* appropriately.**  Round 2 subclause change/ addition: *Staff should be trained to use these skills when communicating with other staff, as well as with patients and families.*  *Service user/NGO comments: ***Strongly support***We see this lacking in practice. Staff must be trained and assessed before handling patients. A bad experience really bruises one for life. The illness is challenging enough to have to deal with staffs that are not well trained and show no empathy to the patients with very little care to begin with. ****  *Healthcare staff comments: ***Validation is an important skill.**** | Mean round 1: 4.7  IQD round 1: 0.0  1 respondents disagree  Mean round 2: 4.8  IQD round 2: 0.0  Consensus reached |
| 1.3 | Round 1 item: **Staff need to pay careful attention to furniture and subtle cues that may make people feel intimidated.**  *In meetings we suggest that the seating should be as close as possible to circular, with no back row.*  *Service user/ NGO comments: ***There should never be a back row. Patients and carers will easily feel intimidated if they are seated in the back row seats. Sitting together will indicate sense of belonging, unity and closeness. All for the same goal.****  *Healthcare staff comments: ***Agreed***It depends on the numbers of attendees. Not many meeting places that can cater for this. Personally I don't think place of seating will significantly affect you.***gives a sense of equality, totally agree with this point.***Not every setting has luxury of space or flexibility to rearrange the seating****  Round 2 subclause change/ addition: *In meetings we suggest that the seating should be as close as possible to circular, with no back row, if space allows.*  *Service user/NGO comments: ***Ok****  *Healthcare staff comments: ***None**** | Mean round 1: 4.4  IQD round 1: 0.6  Consensus reached |
| 1.4 | Round 1 item: **The chair of the meeting should play a facilitator role and take care not to dominate.**  *Service user/ NGO comments: ***Yes, the chair leads. Must be firm but wise and not dominate.****  *Healthcare staff comments: ***any particular criteria of a person to be selected as chair?***Need to overcome overdependence of other participants on the chair***This relates a lot to question 4.1.1.2, with the training, hopefully.****  *Service user/NGO comments: ***Ok***The chair of the meeting plays a crucial role and I do feel the chair would be more than happy to facilitate rather than dominate. Hence, working together being the goal and no veto involved. **** | Mean round 1: 4.4  IQD round 1: 0.5  1 respondents disagree  Mean round 2: 4.7  IQD round 2: 0.5  Consensus reached |
| 1.5 | Round 1 item:  Round 2 item: **The chair of the meeting should be someone who has good meeting skills and skills in listening and validating, understands the topic and the context of the meeting and should be chosen with the agreement of the other members of the meeting. The chair should not be chosen purely on the basis of grade and profession.**  *Service user/NGO comments: ***Everyone has strengths. Rotating would give everyone a chance to lead and recognize the challenges being on the other end too. ****  *Healthcare staff comments: ***A good chance for staffs to practice leadership skills.**** | Mean round 2: 4.4  IQD round 2: 0.5  Consensus reached |
| 1.6 | Round 1 item: **The meeting chair needs to create a non-judgmental, validating environment.**  *They need to ensure that people are not denigrated by other people in the meeting. This is particularly important for more junior staff.*  *Healthcare staff comments: ***Crucial point***The chair must use simple language and not medical jargons which patients or their carers do not understand.***How about giving the more junior staff or nurses/MA to chair the meeting?****  *Service user/NGO comments: Agree, non-judgmental and validating environment will create a positive environment during the meeting as no want feel intimidated and foster a feeling that their opinion matters.***Ok***thank you**** | Mean round 1: 4.3  IQD round 1: 0.5  1 respondents disagree  Mean round 2: 4.8  IQD round 2: 0.0  Consensus reached |
| 1.7 | Round 1 item: **The meeting chair needs to pay careful attention to power imbalances and make a special effort to elicit and validate opinions from people that may be feeling intimidated.**  *Service user/ NGO comments: ***We must make everyone feel they are able to express, share and voice out opinions and suggestions. It must be a fair ground. Patients, carers and junior staff must feel that their voices are being heard and that their opinions matter. The role of the chair is crucial to ensure this happens. ****  *Healthcare staff comments: ***yes ***Chair needs to receive appropriate instructions on this***For that, the chair needs to be a person who is known for being unbiased, and ready to receive comments from subordinates. Again, i feel that giving opportunity to more junior staff will create a more interactive meeting.****  *Service user/NGO comments: ***Ok***The chair needs to be a fair person, why not a patient be the chair too for certain issues especially on things that involve much of their lives. Patients have the most empathy compared to any of the collaborative partners. **** | Mean round 1: 4.5  IQD round 1: 0.5  1 respondents disagree  Mean round 2: 4.6  IQD round 2: 0.5  Consensus reached |
| 1.8 | Round 1 item: **Providing paper to people who might normally feel intimidated can encourage them to express themselves.**  *Service user/ NGO comments: ***This always works!****  *Healthcare staff comments: ***empower them to talk rather than allowing them to feel inferior and unheard***Allow nonverbal expression***True to some extent, but the ultimate aim is for their ideas to be recognized. If a paper is given, the expression would be anonymous. It would be better to train these people to overcome their intimidation, probably through validation, empathy as well as mature coping strategies.***The process is time consuming****  Round 2 subclause change/ addition: *This is more relevant to large staff meetings, where some staff may feel intimidated, even with assertiveness training. Brainstorming sessions, where staff are asked to write ideas and put them in a box can also save time in a large meeting.*  *Service user/NGO comments: ***Agree***Yes, I agree. Slowly they will then gain confidence after seeing how their opinions matter and carries weight in the discussions. ****  *Healthcare staff comments: ***I like the idea of brainstorming sessions, or breaking up in smaller groups to manage tasks.***Agree about this process is time consuming, and empowering them to speak is equally important too.**** | Mean round 1: 4.3  IQD round 1: 0.5  1 respondents disagree  Mean round 2: 4.3  IQD round 2: 0.5  Consensus reached |
| 1.9 | Round 1 item: **Breaking up into smaller groups in larger meetings helps more voices to be heard and allows people to speak that normally feel intimidated.**  *Healthcare staff comments: ***one to one session with the person who feels intimidated in any way to hear their concern***Ya and also try to explore more 'unvoiced needs' issue from patients or their family members. if needed one to one discussion might be encourage.***Less intimidating for the shyer participants***Other than more people speaking, it would be very time-saving.****  *Service user/NGO comments: Yes, it could be one of the solution. However, there must be proper documentation of each meeting, so that we could gather all the information.***Agree***I agree when patients that are involved are more of an introvert and would need some time to warm up **** | Mean round 1: 4.4  IQD round 1: 0.5  1 respondents disagree  Mean round 2: 4.4  IQD round 2: 0.5  Consensus reached |
| 1.10 | Round 1 item: **Staff in leadership roles should be trained in democratic and transformational leadership styles.**  *These leadership styles both emphasize autonomy, engagement, values and internal motivation, rather than leading by using threats and rewards as a form of motivation.*  *Service user/ NGO comments: ***Everyone's opinion matters and must be considered to achieve this goal. ****  *Healthcare staff comments: ***Staff should be allowed the opportunity to attend leadership courses and allow them to demonstrate the leadership skills by assigning them specific tasks.***Essential ***I am not sure whether training would promote such relationships. It is the actual demonstration of desired leadership style that promotes open communications.****  Round 2 item: **Staff in leadership roles should be mentored and trained in democratic and transformational leadership styles.**  *Service user/NGO comments: Agree on this, for those staff whom lacking in this,must be allow to attend training ***Very good***Allowing them some space for them to develop their own leadership style as well****  *Healthcare staff comments: ***Agree on mentoring**** | Mean round 1: 4.6  IQD round 1: 0.5  Consensus reached  Mean round 2: 4.7  IQD round 2: 0.5 |
| 1.11 | Round 2 item: **All staff should be given some leadership opportunities appropriate to their skills and experience. Junior staff should be given opportunities to chair meetings and mentored in this by more senior staff.** | Mean round 2: 4.7  IQD round 2: 0.5  Consensus reached |
| 1.12 | Round 2 item: **The people involved in a meeting should be asked if they have any questions or feedback at the end of a meeting.**  Round 2 subclause change/ addition: *This includes asking patients if they have feedback at the end of a consultation.*  *Service user/NGO comments: Agree on this***TRUE**** | Mean round 2: 4.8  IQD round 2: 0.0  Consensus reached |
|  | Round 1 item: **General comments on autonomy**  *Service user/ NGO comments: Intentionally collect feedback from patients about their experience in the consultation process***Staff should encourage patients to talk more and know that silence means something is wrong.***Encourage questions, opinions and suggestions.****  *Healthcare staff comments: Rotate chair and allow user or carer to chair***There must be strong emphasis on RESPECT for patients in staff training***might need to think of any mechanism to facilitate those who might have problem to voice their view ***Always remember to allow question-and-answer sessions during each meetings. ***Nil***Providing written questions and responses is one way of overcoming intimidation in open dialgoue especially if the hierachial status is wide. ***Giving opportunity to each person attending the meeting to convey their opinion verbally or in a written form.***Make them feel important, make them feel that everyone has equal rights and all opinions and comments should be accounted for.***Very small meeting members to help autonomy of patients/carers. Other members can observe using observation room/cctv**** |  |
| 2.1 | Round 1 item: **Systems should be designed so that there are as few transitions between healthcare providers as possible. If possible patients should see the same doctor on each visit (see appendix).**  *Patients report that they do not like having to tell the same story many times to different doctors and then never seeing them again. If they are unable to form a relationship with their healthcare provider then a therapeutic alliance will not form. Healthcare providers also report that they are more satisfied if they follow up their own patients and most report it is more efficient than seeing patients they have never met. This is particularly important in the early stages of treatment, where illness is unstable and where patients have not yet returned to previous levels of functioning. This may need to be balanced against the training needs of junior staff and service flexibility.*  *Service user/ NGO comments: Very true! I have experience seeing the same doctor from 2011 to 2014 and it really helps me a lot! Now I have to see different doctors at every visit, and I feel lazy to tell my stories again and again. The communication is just superficial, I tell the surface stories and the doctor gives surface suggestions. No chance to explore further. After all, why share so much if I may not see him again?***Agree, some healthcare provided are new to patient case (not seeing their own patient) and don't bother to read patient's case file, here the session is less effective. The patient will feel frustrated***Seeing the same doctor is good for comfortablity of the patient but seeing a different doctor once in a year (for example) is also important as the patient might want to hear some other opinion or they might feel comfortable with the new doctor. But changing doctor on every session is not good either.***Fully agree on this. Patients must see the same doctor (expert and not an MO) until they are stable. This builds trust and also confidence among patients and also carers. Once they are stable, this can be flexible. Patients can then meet other doctors. However, the doctors if possible, must know the history of the patient so that patients don't have to go in depth again about their illness or traumas etc as sometimes this becomes a trigger and really its frustrating and tiring to be telling the story again and again. Also, although the patient is stable, the different doctors that they see must be ones with empathy and knows what to do next (action plan). ***Seeing the same doctor would help build rapport & help patients feel connected to the treatment process. As a patient, I felt truly disconnected from my treatment plan because it was handled by different doctors. After moving to a hospital that assigns patients to the same doctor throughout their treatment process, I began to feel a sense of connection. My doctor knows my story from the beginning, so I didn't have to keep repeating the same story. Repeating my story to different doctors when I was unstable prevented me from seeking help because I had the idea that nobody cares.*  *Healthcare staff comments: Yes. Continuity is important***however this is difficult when human resources (patient:doctor ratio is very wide in Malaysia) are limited and doctors having other duties to fulfill (eg seeing emergency cases, attending administrative meetings etc) ***Yes, the personalize care or patient centre care approach is needed. ***Ideally, the patients should see the same doctor on each visit. In reality, this may not be possible due multiple factors such as training of junior staff, lack of manpower, and other service needs. Good communication is key.***Will be challenging in Malaysian context duke tho limitations in human resource***Agree especially during the early stages of treatment. The 'step-down' process to a different team eg more junior doctor can be done subsequently once the patient is more stable.***I believe this is the most important aspect in building a therapeutic relationship. Without this, it is just going to be another clinic visit, continuing medications and giving another TCA to see another doctor. The new doctor would not understand the dynamics of the patient (as they have many other patients to see), no knowledge on the background history. Any decision taken would be a cross-sectional decision (which is not always good).***Patient will have better compliance with good therapeutic alliance ****  Round 2 appendix change/addition: *Patients report that they do not like having to tell the same story many times to different doctors and then never seeing them again. If they are unable to form a relationship with their healthcare provider then a therapeutic alliance will not form. Healthcare providers also report that they are more satisfied if they follow up their own patients and most report it is more efficient than seeing patients they have never met. This is particularly important in the early stages of treatment, where illness is unstable and where patients have not yet returned to previous levels of functioning. This may need to be balanced against the training needs of junior staff and service flexibility. If patients are not recovering then they should be discussed in supervision or reviewed by another doctor.*  *Service user/NGO comments: Agree***Wholly agreed***Strongly support***Retelling the story is tiring and cumbersome when we could just key in the reports at the end of the day for the respective doctor to then have access to it. It is very challenging to be telling our story when we are unstable, imagine telling our story to a different person every single time. ****  *Healthcare staff comments: ***Relevant and important comments and concerns, which are covered in the modified appendix.***agree***Agree on modified appendix.**** | Mean round 1: 4.7  IQD round 1: 0.1  Consensus reached |
| 2.2 | Round 1 item: **A “primary nurse” system should be used for inpatients (see appendix).**  *This allows inpatients to have one nurse that they are familiar with throughout their stay. This nurse will be responsible for the medium- long term care needs of the patient, including patient education, discharge planning, liaising with other professionals and forming a relationship with the patient’s family. This nurse will not be on every shift, but may see the patient several times per week. We recommend that an “associate nurse” is also appointed for each patient to cover the primary nurse.*  *Service user/ NGO comments: This is good, the patients can have someone to turn to, and to tell everything happened to them.Trust can be enhanced, and thus improving the effectiveness of the therapy.***It makes the patient will 'cared for'***This is important as patients must feel safe, and by having somebody that they are familiar with, helps the patients in their recovery and gives the hospital a good repo as well. However this nurse must be a senior nurse because if the nurse assigned is not fully trained or is not able to handle her tasks well, it will hurt the recovery process too. ***This would help unstable patients feel a sense of familiarity when being warded.*  *Healthcare staff comments: ***It is a good idea to have a primary nurse or "case manager" for inpatients.***May be difficult to implement***Interesting concept especially the fact that the nurse will not be on every shift but may see the patient several times per week. Can be similar concept with a case manager.***What you mean is a designated case manager? This is in accordance with the community psychiatry care setting, yes, a designated nurse should be appointed.***Manpower issue is very common, a significant obstacle to run a new system****  Round 2 subclause change/ addition: *This allows inpatients to have one nurse that they are familiar with throughout their stay. This nurse will be responsible for the medium- long term care needs of the patient, including patient education, discharge planning, liaising with other professionals and forming a relationship with the patient’s family. This nurse will not be on every shift, but may see the patient several times per week. We recommend that an “associate nurse” is also appointed for each patient to cover the primary nurse. Nurses need to be given training on how to fulfill this role.*  *Service user/NGO comments: ***Ok**** | Mean round 1: 4.8  IQD round 1: 0.0  Consensus reached |
| 2.3 | Round 1 item: **Systems should be designed in ways that optimize relatedness between staff (see appendix).**  *Dividing the staff into multiprofessional teams is one way of increasing relatedness between staff and has been shown to significantly improve outcomes (23,24). This is already being done in the creation of community mental health centers. Teams could care for a particular group of patients (e.g. drug users, adolescents) or they could care for a particular geographical area. These teams could include staff members covering different parts of the hospital i.e. staff from wards, clinics and the community would be on the same team. Training needs could be met by rotating staff within the same team, without disrupting relationships.*  *The number of other staff that each person needs to collaborate with needs to be manageable. Staff report that limiting the numbers of medical officers on chronic wards has created a more collaborative atmosphere in Hospital Mesra Bukit Padang.*  *Service user/ NGO comments: ***Ideal but must see whether can be done in hospital setting eg, not enough trained staff***I totally agree. It must be a manageable number in a group too. Big groups can be overwhelming too especially if patients are not stable or if patients are of schizo type and have social anxiety. ****  *Healthcare staff comments: ***this statement is unclear .... "Staff report that limiting the numbers of medical officers on chronic wards has created a more collaborative atmosphere in Hospital Mesra Bukit Padang."***Limitations in human resource will have too be addressed***-****  Round 2 appendix change/addition: *Dividing the staff into multiprofessional teams is one way of increasing relatedness between staff and has been shown to significantly improve outcomes (23,24). This is already being done in the creation of community mental health centers. Teams could care for a particular group of patients (e.g. drug users, adolescents) or they could care for a particular geographical area. These teams could include staff members covering different parts of the hospital i.e. staff from wards, clinics and the community would be on the same team. Training needs could be met by rotating staff within the same team, without disrupting relationships.*  *The number of other staff that each person needs to collaborate with needs to be manageable. Staff report that limiting the numbers of medical officers on chronic wards has created a more collaborative atmosphere in Hospital Mesra Bukit Padang.*  *Service user/NGO comments: ***Ok****  *Healthcare staff comments: ***No comments.***yes**** | Mean round 1: 4.9  IQD round 1: 0.0  Consensus reached |
| 2.4 | Round 1 item: **Representatives should only be sent to meetings when they are aware of the issues or are planning to join a committee in the long term.**  *Service user/ NGO comments: ***Yes of course. These are serious matters and require serious people involved. Those that have the endurance and want to go the long haul.****  *Healthcare staff comments: ***anyone can join the meeting as long as they are working in the department***yes and able to discuss the issue with their knowledge and skill***Representatives are able to contribute and can benefit from the meeting if they are aware of the issues beforehand. Sending unrelated representative may be a waste of resource, as they may be able to contribute to other services or tasks instead.***However, the right person may not always be available***Do not understand the statement. What Committee ?? When does this come in?***Or at least they receive some briefing prior to the meeting!***If someone is unable to attend the meeting, there is no point in sending a substitute with no clue as to what is being discussed. This holds true for all levels of professionals, including specialists. Head of department should not randomly choose who is to attend. Equal opportunity should be given to everyone in the department and they are given opportunity to choose to attend meetings that they feel is appropriate to them.***They should consistently join meetings, in case of improvised issue****  Round 2 item: **Representatives (people that represent longer term committee members) should only be sent to patient care planning meetings or other hospital meetings when they are aware of the issues or are planning to join a hospital committee in the long term.**  *Service user/NGO comments: ***Ok***Representative of the patients? They should be in the committee meeting for the long term. They must be in for the long term and must be in the know. ****  *Healthcare staff comments: ***More clear statement after modification.***ok***Agree**** | Mean round 1: 4.4  IQD round 1: 0.5  Consensus reached |
|  | Round 1 item: **General comments on Relatedness**  Round 2 item: **0**  *Service user/NGO comments: ***Ok****  *Healthcare staff comments: ***Agree**** |  |
| 3.1 | Round 1 item: **All staff should be trained in the following areas: [insert list**  *Service user/ NGO comments: ***Each staff ( especially junior staff) should be empowered to work alongside more senior staff. ***Staff must be well equipped with the required skills particularly those working in the psychiatric department. ****  *Healthcare staff comments: Most staff in malaysia have limited training***Need to reduce the gap between the doctors and paramedics/allied health in terms of authority in decision making. ***I believe training should be given to more junior staff, nurses and MA to make them aware that their opinion is important. Training should incorporate ways to improve their confidence and avoid feeling that they are of lower professional status. In the training, everyone should be addressed with a similar notion, to avoid making one feel inferior to the other.***Effective communication skills should be included in staff training****  *Service user/NGO comments: I believe it is good that training provided to junior staff and to treat junior staff's opinion as important as senior staff.This will reduce inferiority of the junior staff and boost their confidence***Very good****  *Healthcare staff comments: ***No further comments***Agree with comments***Capacity to provide training also depends on level of workload, manpower and budget. Other than training, effort to practice these skills as part of work culture should also be done. **** | Consensus reached |
| a | Round 1 item: Staff should be trained in **Interprofessional working** | Mean round 1: 4.6  IQD round 1: 0.5  Consensus reached |
| b | Round 1 item: Staff should be trained in **Meeting skills** | Mean round 1: 4.6  IQD round 1: 0.5  Consensus reached |
| c | Round 1 item: Staff should be trained in **Assertiveness skills** | Mean round 1: 4.8  IQD round 1: 0.1  Consensus reached |
| d | Round 1 item: Staff should be trained in **Validating other people’s opinions and giving feedback** | Mean round 1: 4.8  IQD round 1: 0.1  Consensus reached |
| e | Round 1 item: Staff should be trained in **Reflective practice** | Mean round 1: 4.6  IQD round 1: 0.5  Consensus reached |
| f | Round 1 item: Staff should be trained in **Collaborative decision making and problem solving** | Mean round 1: 4.6  IQD round 1: 0.5  Consensus reached |
| 3.2 | Round 1 item: **Training in collaborative competencies should be skills based and include role playing sessions and reflective components.**  *Training is based around the principles of interprofessional education where staff learn from, with and about one another’s roles.*  *Service user/ NGO comments: ***This definitely strengthens the team, builds respect towards each other's expertise and knowledge and builds unity. ****  *Healthcare staff comments: ***also hands-on training under supervision***Nil***-**** | Mean round 1: 4.5  IQD round 1: 0.5  Consensus reached |
| 3.3 | Round 1 item: **Most nursing and other professional staff working in psychiatric institutions should either have post-basic training in psychiatry or be undergoing this training.**  *Recognition needs to be given that psychiatric nursing is a specialized field and requires the application of multiple higher level skills.*  *Service user/ NGO comments: ***This is crucial as to ensure staff in psychiatric institution don't judge nor stigmatize patients. And also ensures that patients are treated with care, understanding and full of empathy.****  *Healthcare staff comments: ***Staff should also have the passion and dedication to work in the psychiatry field.***Training opportunities are limited***Also, it must be emphasized that there should be equal distribution of staffs that are sent to the psychiatry department. I have worked in places where when a person has disciplinary issues, they are sent to work in the psychiatry department. The stigma must be abolished.****  Round 2 item: **Most nursing and other professional staff working in psychiatric institutions should be interested in working in psychiatry and either have post-basic training in psychiatry or be undergoing this training.**  *Service user/NGO comments: Yes, agree they need proper training especially on being non-judgmental toward patient. As patient, I have met some nurse whom are judgmental and look down on mental illness patient and giving negative remark. I think if the nurses have an interest to work in psychiatry this incident can be reduce.***Good***This is of utmost importance as currently there has been a lot of complaints of mistreatment of patients in psychiatric wards. Nurses and staff members are very rude and treat patients without empathy and proper care nor concern. Hence patients become very traumatized and the experience triggers them further and worsens their condition at times. ****  *Healthcare staff comments: ***Anyone working in the psychiatry department should have the passion, commitment and dedication in order to provide the best care for patients.***that is very important***Agree**** | Mean round 1: 4.8  IQD round 1: 0.0  Consensus reached |
| 3.4 | Round 1 item: **Staff should be mentored. Staff with post-basic psychiatry training can mentor staff that do not have post-basic training.**  *Staff working in psychiatric environments need regular supervision meetings, which are reflective and supportive.*  *Service user/ NGO comments: ***Yes. Support for staff working in the psychiatric environment is important. It can be very challenging to manage psychiatric patients on a daily basis. Staff must be well supported and given encouragement. Keeping the morale of staff positive is important as we need staff to be constantly motivated and happy in executing their jobs.****  *Healthcare staff comments: Post basic training in malaysia is grossly inadequate. Minimal skills taught***Human resource limitations. Insufficient mentors***Mentally ill patients are special population with special requirements and needs unlike physically ill patients. The paramedic training syllabus unfortunately does not provide much training in this aspect thus the need for post basic learning and mentoring system. ***-****  *Service user/NGO comments: Staff whom work in psychiatric setting should be given debriefing when needed especially the junior staff, the case is psyhiatric setting sometimes can be overwhelming***Good****  *Healthcare staff comments: ***No further comments.***Agree with comments***Agree on regular supervision meetings.**** | Mean round 1: 4.9  IQD round 1: 0.0  Consensus reached |
| 3.5 | Round 1 item: **Specific staff should be allocated to work in psychiatry in district hospitals and primary care, to allow these staff to develop the required competencies.**  *These staff can be mentored by staff with post-basic psychiatric training.*  *Service user/ NGO comments: ***indeed. Anyone working in psychiatry must have the required competencies. It is really challenging for those not having the required skills, knowledge and understanding to deal with psychiatric patients and their families. ****  *Healthcare staff comments: ***Limitations in number of staff avsilanle to do this***Agree but most often, post-basic psychiatric trained personnel are not available at primary care or district level hospitals***However, need to strengthen the number of staff with post basic training first.***-****  *Service user/NGO comments: Strongly agree***Why not include the training as a compulsory subject in the current nursing syllabus? We need to make best use of existing resources. Engage a qualified trainer to train. Make this agenda urgent and important, not something to be done later when we "have more resources". Who knows, that time will never come. Instead of waiting passively for something to happen, why not we just start from one? Even a small start like engaging one trainer will start the ball rolling.***crucial****  *Healthcare staff comments: ***Properly trained staff at primary care and district level is important to provide accessible mental healthcare to those living in rural areas. These staff can also collaborate with villagers and community leaders in outreach programs, education and community psychiatry services.**** | Mean round 1: 4.6  IQD round 1: 0.5  Consensus reached |
| 3.6 | Round 1 item: **Higher authorities in the health service should review the skills mix.**  *There is a particular shortage of some groups of allied health staff, which makes interprofessional collaborative practice difficult.*  *Service user/ NGO comments: ***i think this is still lacking in Malaysia***It must be balanced to reflect a good and positive result. ****  *Healthcare staff comments: ***namely clinical psychologist and social workers***Nil***The mismatch is sometimes due to personal (staff) commitments (like they want to be where the family needs them to be rather than where service needs them to be). Hence, it is always difficult to balance the mismatch between needs (gaps) and available resouces. This is a system as well as social problem.***Again, similar to my comments in 4.3.1.3. Before educating the public, we must educate healthcare professionals that psychiatry is a specialized feel for nurses as well. We should try to get more people in.****  Round 2 item: **Higher authorities in the health service should ensure that there is an appropriate skills mix, i.e. that there are adequate numbers of all professional groups, including clinical psychologists and social workers.**  *Service user/NGO comments: Agree***Good***I agree plus we must have peer support specialist (recovering patients/survivors) in this collaborative practice to provide insights and help improve the mental health arena as a whole. I strongly believe that this is the missing link that must now be inserted for the collaborative practice to make it complete. Without inputs from patients the solution can never be complete. ****  *Healthcare staff comments: ***Yes, we are still lacking in numbers of clinical psychologists and social workers.***...and counsellors at the primary level***Agree ***Agree. Effort to convey the message has been consistently done to alert the higher authorities.**** | Mean round 1: 4.6  IQD round 1: 0.5  Consensus reached |
| 3.7 | Round 1 item: **The use of the Ministry of Health's “Patient’s Unvoiced Needs” program, is recommended.**  *A video is shown in the waiting room, which encourages patients to write down what they would like to discuss with the doctor before the appointment. (Copy and paste link: file:///C:/Users/307252456/Downloads/Reducing_Patients_Unvoiced_Needs_-the_Ma.pdf)*  *Service user/ NGO comments: ***Cant view the link ***It gives the patient an idea on what and how to share things. And by writing down their thoughts it helps them to organize their thoughts, declutter whats in the mind and build confidence on delivery. ***This would be a really good implementation. Patients often forget what they planned to say after waiting for a long time to see their doctors because they're tired.*  *Healthcare staff comments: ***yes to facilitate and also for those we are too shy to voice their issue/concern verbally. This is also facilitate the healthcare provider to understand patients 'thought' and allow them to probe more into patient's concern. ***Nil***Have read report of the study***Challenging to implement for patients with very poor cognitive ability or poor literacy.****  Round 2 subclause change/ addition: *A video is shown in the waiting room, which encourages patients to write down what they would like to discuss with the doctor before the appointment. This was introduced because it was discovered that many patients do not discuss what they intended to discuss with their doctor.*  *Service user/NGO comments: It is a good strategy***Ok***I do feel that this is because the doctors are not able to build repo/ trust with the patient because situations and patience experience with doctors are rushed and to be fair doctors have a lot on their plate. But more PR skills and empathy can be shown during this very chopr perod. ****  *Healthcare staff comments: ***Good idea, although it could be challenging and unsuitable on patients with poor cognitive function and poor literacy.***Agree with the statement in new appendix***Agree**** | Mean round 1: 4.6  IQD round 1: 0.5  Consensus reached |
| 3.8 | Round 1 item: **Each patient should have a written care plan, which they can share with all people involved in their care.**  *This would be produced at care planning meetings attended by the primary nurse, primary doctor, patient, family and all others involved in their care.*  *Service user/ NGO comments: Ya, this is good.***The hospital should provide guidance on how to do this***Written care plan can also be produced with the present of staff and patient only if the time is limited.***This is a very good idea as all involved are updated with the right news. ***This is important for patients to see their progress and gives them a purpose; something to look forward to and it will drive and motivate them to achieve their goals. ***Very good and important, especially for carers. They need to know what's the plan for their loved ones and what they can do to help. This written care plan can act as a roadmap to patient's recovery.*  *Healthcare staff comments: Important for plan to be developed with patients and carers***A practice done only verbally by the staff/doctors.***not necessarily everyone should be there as it can be tedious to make sure everyone is around at the same time. it is a team effort so a represenattive of the treating team should be enough***yes the personalise concept should be applied***A small notebook can be used for this purpose which patient can easily carry around.***May be an issue with patients losing their care plans***Care plan is as far as the healthcare system covers. Eg. if a patient desires to be able to go to the gym once a week, can this be accommodated? It must be withing the scope of service available. ***We have been doing this (care plan for patient) but unfortunately it is not in a written form for the patient to keep and refer. Something that is workable.***We must be cautious in this matter. Some could use the care plan for other reasons such as insurance claim. I would not agree completely on this because all documents are private and confidential. With this, can we assure confidentiality is maintained?***Can increase efficiency of care plan****  Round 2 item: **Each patient should have a written care plan, which they can share with all people involved in their care.**  Round 2 appendix change/addition: *A care plan is a list of actions which will help the patient move towards recovery goals and staying well. This can include actions provided by healthcare professionals (e.g. prescribing medication), actions done by the patient themselves (e.g. exercise) and actions done by family members (e.g. listening if the patient wants to talk). Writing the plan is a way of making sure that all members of the team, including the patient and family, know what the plan is and are working together towards the same goals. The complexity of the plan depends on the needs of the patients and a simple plan may just consist of a handwritten list of recovery goals and actions written in the patients clinic notebook.*  *Service user/NGO comments: Agree, good approach but must not forcing patient whom are in unstable state as they might feel overwhelmed by this. It is beneficial those patient whom are more stable***Why not we leverage on technology? Instead of producing a paper notebook to record all these, invite software company to create an app that all stakeholders can use and view and monitor each other. Patient can have individual account in the app which allows access from psychiatrist, psychologist, primary nurse, case manager, caregiver, and related community members. Patient can control who to see what (privacy control). And each stakeholder can cross check and follow up with each other.****  *Healthcare staff comments: ***Modifications have addressed the concerns raised.***ok***A standardized care plan will make the process much easier **** | Mean round 1: 4.6  IQD round 1: 0.5  Consensus reached |
| 3.9 | Round 1 item: **Education and support groups should be set up for patients and carers.**  *Service user/ NGO comments: This is important but the hospital doesn't see its importance. (I was a psycho-education officer for 2 months but cannot continue after the hospital Director is transferred. It seems that the hospital doesn't think it is important.) However, even with such thing, patients are not interested to join too, because of time, distance or they don't think it is important. Maybe they think medicine alone is enough and they don't care to do too much.Good***Agree since the carer is exhausted due to lack of knowlegde at time***Talks or meetings can be held for careers and patient to know each other and sharing of information of how carers are dealing with the patients, so other carers can apply it too.***This allows both sides to be heard without misinterpreting the message.***Support plays a crucial role in recovery. Not only patients must be supported but carers as well as they too struggle, feel lost and feel helpless.***Education is key for patients and carers to understand the illness and that recovery is possible. The stigma in society is very strong, so by educating the patients and families, it will help to empower and strengthen them to recover, and not be brought down by the stigma.*  *Healthcare staff comments: Should be peer lef***support group for patients is hardly any in Malaysia***...as an NGO that is run by them***Yes, i have seen this in Netherlands (for PTSD people) ***There is a need for education and support group for patients and carers in Malaysia, which is currently lacking especially in smaller towns. Social media can be a platform for education and support as it is popular, widely used and easily accessible.***Resource scarcity will be the limiting factor***Need leaders for support groups***Psychoeducation program is a practice in majority of hospital nowadays. However, I foresee that support group is going to be very challenging. Too much for hospital/primary care to handle is the. Collaboration with NGOs probably the way to go.***It is already there, just that they are not aware. Rather than setting up, probably can educate them on the options available.***Education and support groups will increase compliance and reduce readmission.****  Round 2 item: **Education and support groups should be set up for patients and carers, including groups led by patients and carers. Brochures and promotional materials about existing groups should be made available in clinics and wards to ensure that patients, carers and staff are aware of their existance.**  *Service user/NGO comments: This support group is essential. I previously had asked Malaysian Mental Health Association on this, they have done before but cannot continue as most patients did not turn up for the session after attending some of the session.***Wholly agreed. Having the promotional materials readily available would be really helpful. Perhaps also put some books there, eg collection of patients' recovery stories. ***Good.***How do we provide this? There must be a central registry that we as MIASA can contact and can then provide the input and materials where doctors can help disseminate or put up. ****  *Healthcare staff comments: ***Nothing further to add at this point.***agree***Agree**** | Mean round 1: 4.8  IQD round 1: 0.0  Consensus reached |
| 3.10 | Round 1 item:  Round 2 item: **Patients and carers who are able and willing to help others should be trained to work as peer support workers and educators.** | Mean round 2: 5.0  IQD round 2: 0.0  Consensus reached |
| 3.11 | Round 1 item:  Round 2 item: **Peer support workers and educators should be paid an honorarium for the time spent doing the work.** | Mean round 2: 4.4  IQD round 2: 0.5  Consensus reached |
| 3.12 | Round 1 item:  Round 2 item: **Education for both the public and professionals should involve patients and carers as educators.**  *Service user/NGO comments: Agree on this***TRUE**** | Mean round 2: 4.8  IQD round 2: 0.0  Consensus reached |
| 3.13 | Round 1 item: **Written materials should be available in doctor’s rooms for patient and carer education.**  *Service user/ NGO comments: Good. Can prepare written material for distribution. Great help for the patients and carers who would appreciate.***I strongly agree as patient need to be educated on their illness by providing resources***Written materials can be flyers, books or a simple posters with mental health informatiom.***This allows patients that have trouble vocalizing their problems/needs to have a material that they can point to the psychiatrist on which area that is troubling them. ***Indeed. This way, it helps the patients and carers to equip themselves with the relevant knowledge***It'll be good to include information in graphical forms (eg: infographics) and not lengthy text.*  *Healthcare staff comments: To be developed by professionals n peers***or at the waiting area***..including materials given for free by pharma company***Yes***Written materials help strengthen the retain of information and patient or carer can read them in their own time, at home.***Creation of such references will be challenging***Better to be placed in waiting areas. Time with the doctor is limited and patients/caregivers will not have the time to browse through education materials. Any material in the doctor's room should be reference materials for their own use only. ***We can divide the tasks (prepare certain chapter) and distribute to the other institutions***-***Electronic written materials would be more flexible and accessible****  Round 2 item: **Written materials should be available in doctor’s rooms or waiting room for patient and carer education, which should also be available on line. Patients and carers should be invited to write some of these materials if they are interested in doing this.**  *Service user/NGO comments: Very beneficial and advisable***Yes. And please reach out to NGOs as we have recovering patients who can write these materials as reference for other patients. Let's make recovery a holistic process that is beyond the hospital building.***There are so much mental health related info in internet already. If the patient and caregiver really have the heart to find out more, they can actually look online, no need to rely on paper material. After all, after they read it, they will just keep it aside or throw it away. Why waste paper? And it is not really hard to produce the material. I have visited Singapore Simei Care Centre(a psychiatric rehabilitation centre). They have so much resources available. We can just work with them. Locally we also have MMHA who already published a lot of good material. Why not we just make the best use of existing resources rather than reinventing the wheel.****  *Healthcare staff comments: ***No further comments.***agree**** | Mean round 1: 4.7  IQD round 1: 0.1  Consensus reached |
| 3.14 | Round 1 item: **Information displayed on the wall of the clinic should be related to mental health.**  *Service user/ NGO comments: Good. Instead of wasting time staring into a blank wall, why not put on some useful information that may help?***Yes, but in friend;y manner***The info on the wall should not show about type of mental illness as the patient might feel overwhelmed with too much info and feel like he is very sick. It can be as simple as a motivation words or a pleasing positive photo.***Allows patients to identify and understand what they are going through***Talking about mental health and getting help is still a taboo in Malaysia, hence having info on the walls would greatly help. The public must understand that there is no health without mental health too. We need to be aggressive in promoting mental health awareness as those with mental illness has increased drastically and those that have committed suicide is increasing at an alarming state too. Hence, everyone should be on the same page and we need everyone's help to manage this growing concern. ***Especially on the possibility of recovery and various NGOs or services that they can contact to get more support.*  *Healthcare staff comments: In clear understandable language***not done in most facilities***infographics***the contain should be carefully written to avoid any stigmatize ***Posters in the form of infographics are an attractive way to relay information.***Need suitable resource***Mental Health is very broad and encompassing. Healthy lifestyle is also mental health. ***1. Can work with Unit Pendidikan Kesihatan in hospital*  *2. Can organize some competition in order to have more materials (eg infographic poster competition on depression) ***Not only information on signs and symptoms, but also information on how well can someone with mental illness live. Can put up information about people with mental illness who is socio-occupationally high functioning. ***Related pamphlets and help care line should be displayed too ****  Round 2 item: **Information displayed on the wall of the clinic should be related to mental health, particularly ways to improve mental health and well being. Information displays need to be clear, positive and sensitive to what patients may find distressing.**  *Service user/NGO comments: Strongly agree that the material must be carefully designed and did not downgrade the patient***Yes please on healthy lifestyle, high functioning patients that they can take inspiration from.***Again, it is not hard to find all these material. Just search online, there are so much. Put up something that people can't find on internet. Suggest to have some interactive content using technology, like a touch screen board that visitors may do a simple and quick mood assessment, find local NGO/support group and even directly contact them, and some other interactive features.****  *Healthcare staff comments: ***Yes, information in maintaining a healthy lifestyle is also good - such as relaxation and stress management techniques. ***definitely!***Get involvement from Patient Education Unit in each hospital. **** | Mean round 1: 4.6  IQD round 1: 0.5  Consensus reached |
| 3.15 | Round 1 item: **Mental health education videos should be shown in the waiting area of the clinic.**  *Service user/ NGO comments: Good. Videos capture people's attention. Since they have to wait anyway, good to let them absorb some useful information.***if possible***This education video is meant for the public who are not mentally ill and not only aim the mental health patient.***Its a effective way to educate those who are at the waiting area***It depends on what kind of education you will be giving? It must be a message of hope always. ***While waiting, patients and families can spend their time watching good videos on mental health. I recommend videos that cover topics on hope, recovery, coping with patients' common struggles, and addressing common stigma (eg: mental illness equals to poor faith, crazy, unstable, etc).*  *Healthcare staff comments: On recovery***we need the videos***basic coping skills, success stories of patient***similar to above comments***At the moment, general health videos are shown in my clinic. Mental health education videos should be shown as well to make it "wholesome". There are still a lot of unfamiliarity and misunderstandings related to mental health issues. These videos would provide a good education opportunity.*** Need local resources***If waiting area is too crowded and noisy, education videos are ineffective. It is useful in quiet and more private environment, or made available (accessed ) through their personal handphones. ***As above***-***A very good approach for indirect psychoeducation and awareness****  Round 2 item: **Mental health education videos should be shown in the waiting area of the clinic as well as being available online. These videos should show positive, hopeful, non-stigmatising views of mental illness.**  *Service user/NGO comments: Yes, to provide is very useful. For example content could be on coping mechanism and real case success recovery story***There are so many mental health videos that can be found online...perhaps you want to create some more customized to local needs? Like make them available in different languages since most of these online videos are in English.***awesomeness! one way of educating the public****  *Healthcare staff comments: ***Videos shown in the waiting room are a good idea.Online videos are also a good idea as they are accessible anytime.***agree***Agree**** | Mean round 1: 4.6  IQD round 1: 0.5  Consensus reached |
| 3.16 | Round 1 item: **A resource room should be available near the waiting room, which contains educational materials (brochures, books, videos).**  *This should be staffed by someone capable of giving education to patients and carers, e.g. a staff member or patient educator.*  *Service user/ NGO comments: This is good! Consultation by a patient educator can be given to those who come to this resource library. Patients and carers can gain extra help that the doctor cannot gives. When I worked as the psycho-education officer, I found that patients actually like to share a lot with me-their success and failure in their journey of recovery...and I can offer help that they previously do not know.***a 'stable patient/ can be assigned as voluteer***This is important to the patient and carers as sometimes the doctor don't have very long time to talk more about the illness, especiaply when they have a lot of patients.***Patients and carers can get a better overview of the psychiatric system (if they are new) ***With knowledge can only recovery happen. Having these kind of materials would be really helpful as sometimes patients are shy or embarrassed to get books on these from bookstores and end up surfing things online that might not be accurate. ***Can also recruit recovering patients to staff the resource room. This not only help the patients and families, but also the recovering patient (patient empowerment).*  *Healthcare staff comments: Also by peers***If staffing is an issue, probably open the room at certain hours eg during clinic days***it can be part of the cubicle in the waiting area without a dedicated staff***yes but depending on the task of staff member with the limitation of medical staff, the function has to be discussed in details. ***Some centres may have limited area for a specific resource room in the clinic. A dedicated space or psychoeducation session conducted by staff can also be considered.***Space and equipment will be limited***Resource materials must include services provided by others outside the primary provider (eg NGO providers).***The staff may be an issue but we can always provide the name or contact number of person that the patient/carer can reach to get more information.***-***Educational materials should be placed in waiting area****  Round 2 item: **A resource room or area should be available near the waiting room, which contains educational materials (brochures, books, videos). This should be staffed by someone capable of giving education to patients and carers, e.g. a staff member or peer educator.**  *Service user/NGO comments: Yes, patient educator is a good suggestion since staff resources is quite limited. ***Love the term "peer educator"***In UK, they have a mental health "Recovery College" where patients can register as students and attend classes for free. Classes can include psychoeducation, coping skills, how to give peer support etc. They have a series of very good and structured curriculum which helps the patients a lot. In every class there are 1 main instructor who is a professional and another facilitator who is a peer. This is something we can model after in Malaysia. You may contact me if you want to be connected to Ms. Sandra from UK who presented this idea and shared her real life experience last time in Hospital Permai.***Having recovering patients mange the resource room would be a great way to empower patients and have them champion their own cause in which is inclusion and lived experience. ****  *Healthcare staff comments: ***Good, nothing further to add.***agree**** | Mean round 1: 4.3  IQD round 1: 0.5  Consensus reached |
| 3.17 | Round 1 item: **We recommend that patients be given a clinic book.**  *This book can be used for the following: Individualised care plans, relapse prevention plans and education, psychological work – e.g. CBT formulation, pages to write down things that they would like to discuss with the doctor (as part of 4.3.1.8)*  *Service user/ NGO comments: This is good! What people talk, they may forget. But when they write down, it is easier for them to remember. And good for other stakeholders to be aware of what care plan is carried out by the patients.***I believe this is helpful since in the hospital patient did not meet the same psyc***This is important as sometimes patient always forgot what to say in the room. They also sometimes forget what the doctor said during the meeting, especially when they meet up once in every 3 months or longer.***An effective way to keep track on issues that can be brought up to the doctor ***Writing things down is a good way to reflect and remove clutter in our brain. It helps to get ourselves organize and gives us something to look forward to. Ultimately, patients will then see hope. ***Strongly agree*  *Healthcare staff comments: Include peer support***this has been practiced in developed country (Australia and UK)and has been shown to be very effective (eg Optimal Health Plan in Melbourne)***patient write down advices given by the healthcare professionals and paramedics but not the details of illness/HOPI or medication used***just concern about how to handle the confidentiality ***This will help empower patients to take part in their own care and management.***Nil***Care Plan should include recovery goals***Budget is an issue seriously. Yes, we can prepare and the patient can purchase from us (cost price ).***This is really good. This can help them express what they can't do in clinic. This will be a constant reminder to them, a kind of CBT workbook.***Very good initiative for better treatment plan****  Round 2 item: **We recommend that patients be given a clinic book. This book can be used for the following: Individualised care plans, recovery goals, relapse plans, education, psychological work – e.g. CBT formulation, pages to write down things that they would like to discuss with the doctor.**  *Service user/NGO comments: Agree***Again, this can be done through an app compared to a paper clinic book. (See my previous comment at 4.3.1.9) Patient's case file is so thick already with only doctors' handwritten notes. A thick clinic book is bulky to carry around and patients may forget to bring. I suggest this app to allow access from those other stakeholders that the patient can hold accountable with in terms of his recovery progress, with patient setting the privacy control. This is an empowerment for the patient. But I am not sure how much patient can commit to this no matter it is in a paper or app form. Maybe applicable to high functioning and mild symptoms patients. For those seriously impaired, maybe caregiver can register as an user to the app.*  *It is also useful for the hospital's patient database management system as everything can be digitalised, thus reducing manual/tedious admin workload previously done by the hospital staff. Save paper as well.*  *Patient data can be accessed by other medical team members in other hospitals if patient move to a new place, or need to be transferred due to certain reasons.***this is awesome and it would be good if patients can also state who can read this book as well. In an event when the patient relapses, a family member or the trusted person that the patient has assigned in the book, then can see what was requested by the patient. This help caregivers in their journey to help as well. ****  *Healthcare staff comments: ***Strongly agree.***Agree on modified content**** | Mean round 1: 4.7  IQD round 1: 0.1  Consensus reached |
| 3.18 | Round 1 item: **Staffing calculations and rotas should take account of the time needed for collaboration.**  *Time needed for collaboration includes time for phone calls to other healthcare staff or families, time for specialists to provide consultation to medical officers, as well as time needed for meetings.*  *Service user/ NGO comments: Good. These are necessary and should be considered into the working hours.***agree***A too short meeting usually cannot dig down what happen to the patient in their everyday life.***Yes this is important***Indeed. Time is an important component in this collaboration.***No further comments*  *Healthcare staff comments: Essential with patient impt***It would be good for time spent caring for patients to be documented to the smallest detail to reflect the workload accurately.***working as a team can reduce the need of the said statement***no comment***Teamwork and communication between staff are essential in this matter.***Limitations expected***Agree but need data to back-up - what norm to use. ***Need to find ways to make it as cost effective and time efficient as possible.***-***agreed**** | Mean round 1: 4.4  IQD round 1: 0.5  Consensus reached |
| 3.19 | Round 2 item: **Topics of discussion should be limited to the things that concern most of the people attending the meeting.**  Round 2 subclause change/ addition: *If staff are unable to contribute at the meeting, or learn from it, then their need to attend should be reviewed.*  *Service user/NGO comments: Agree***Agree***Give staff a couple of chance as it may take them a bit to digest and chew on what is being discussed unless we can send everyone a circular ahead of time so that they can give it a good thought before the meeting. ****  *Healthcare staff comments: ***Agree with the modifications.***agree with comments***Agree on modified content.**** | Mean round 2: 4.5  IQD round 2: 0.5  Consensus reached |
| 3.20 | Round 1 item: **Care needs to be taken in deciding how to use multi-professional meeting time.**  *Topics of discussion should be limited to the things that concern most of the people attending the meeting. If staff are unable to contribute at the meeting, then their need to attend should be reviewed.*  *Service user/ NGO comments: Good. Only those who are involved should attend the meeting.***agree***Too many faces that are passive during the meeting can cause the feeling of intimidated and judged.***Agreed as the time of this staff (that could not contribute) could be better used at other places such as training or patient education. ***We need to give them a few chances however, if they still do not contribute then their need to attend should definitely be reviewed as these matters that are being discussed are crucial and we need everyone's constructive outputs. ***No comments*  *Healthcare staff comments: Discussion made before meetings and meetings used to endorse decisions***It should be okay fro staff who want to learn to also attend the meeting***good to retain the best staff only and phasing out staff that is not really interested in care plan***ya agree***Unrelated topics should be discussed at another time or meeting with the person involved. Non-contributing staff may actually be beneficial for other tasks or services during the meeting, therefore should not waste time with unnecessary attendance.***Best use ood time will be challenging***Should define what kind of problems need to be brought to such multi-professional meetings. Only relevant people need to attend the meetings, which can vary. Hence, the consituents of members at such meetings vary depending on what is to be discussed.***Agree***Staff who are unable to contribute SHOULD NOT attend the meeting. Discussion should always be focused on the issue that is pertinent to the care plan.No other matters should be discussed, including department related matters.***Staff attendance should be flexible, following the priority****  Round 2 item: **Each member of staff should participate in only a limited number of hospital committees and junior staff should sometimes be appointed as committee members.**  Round 2 subclause change/ addition: *This will reduce the load on more senior staff and empower more junior staff.*  *Service user/NGO comments: No further comment***Ok***Capable Junior staff can be appointed with prep work and supervision by senior staff during the first few meetings. I am just concerned because in practise many MOs do a horrible job for example when meeting up with patients and creates trauma for the patients experience and hinders patients from getting the help and treatment needed. ****  *Healthcare staff comments: ***No further comments.**** | Mean round 1: 4.4  IQD round 1: 0.5  Consensus reached |
| 3.21 | Round 1 item: **Each member of staff should participate in only a limited number of hospital committees and junior staff should sometimes be appointed as committee members.**  *This will reduce the load on more senior staff and empower more junior staff.*  *Service user/ NGO comments: Empowering the junior staff can help them to learn faster, and reduce the workload of senior staff.***i'm not sure the practicality of this in the actual setting, do we enough staff ?***Too much participation in hospital committees can deviate the focus and skills empowered.***And this allows junior staff to gain more knowledge and experience ***Junior staff are not experienced and may have a negative impact decision making etc. We can empower the junior staff in other areas but not on crucial decision making etc as they are not experienced and not knowledgeable enough for these areas. Empowerment can be in a variety of ways. ***No further comments*  *Healthcare staff comments: Meetings should be limited and only done when needed***This will give opportunity to gain experience for the junior staff, and time to focus better for senior staff***multitasking is not helpful***Also for grooming the junior staff***There should be a balance of senior and junior staff in hospital committees so that everyone has the opportunity to learn from each other. At the same time, senior staff will not be overwhelmed with too many commitments.***Valid***Junior staff must be prepared and briefed before being sent to attend meetings on behalf of departments. Often, they are sent without prior briefing and cannot contribute anything at the meeting. It must be relevant people, not a matter of being senior or junior. ***And provide guidance to the junior staff even though the more senior staff is not in the committee.***This is important to avoid burn-out as well. ***A limited number of staff is unavoidable, almost in every setting****  Round 2 item: **Better physical resources are likely to improve collaborative practice**  Round 2 subclause change/ addition: *– e.g. adequate inpatient infrastructure, interview rooms, rooms for group sessions, meeting rooms, IT facilities.*  *Service user/NGO comments: Better resources are crucial, but budget could be one issue. Maybe start small first***We may not necessarily use the space in the hospital premise. What is important here is the people. Sometimes doing discussions outside hospital setting can give a fresh outlook and spark creativity and innovation. There are other public spaces that we can use for free, like public library or university library. We can even do it at cafe or restaurant, which provides a more relaxing atmosphere, leading to the production of fresh new ideas.*  *Sometimes people have negative impression with hospital, especially mental hospital. They may feel intimidated/ashamed to come to mental hospital. We can do it at other spaces which are conducive and public will be more willing to get involved.***more color please and lets try and spray some ambipure in the rooms. It doesnt have to smell that way. Its 218 guys!****  *Healthcare staff comments: ***No further comments.***agree***Strongly agree**** | Mean round 1: 4.6  IQD round 1: 0.5  Consensus reached |
| 3.22 | Round 1 item: **Better physical resources are likely to improve collaborative practice**  *– e.g. adequate inpatient infrastructure, interview rooms, rooms for group sessions, meeting rooms, IT facilities.*  *Service user/ NGO comments: True.***Hoping that we can achieve this in future***Comfortable environment can boost productivity and creativity among staffs.***In government hospitals without psychiatric ward, patients are warded together with other patients. This is not an ideal condition for the patient. ***Yes definitely! We need the space for sure. We want patients to feel happy, safe, comfortable and not the typical hospital type ambience :( Patients already feel terrible, let's not add more to that. ***The look and feel of the rooms also play a role in patients' mental well-being, eg the colours of the walls, furniture, etc.*  *Healthcare staff comments: Impt to have access to rooms***Rooms for group session should be plentiful in a psychiatric facility.***multifunctional room is an easier alternative***agree***With good physical resources, a variety of activities can be organised and conducted which eventually can benefit the patients and carers. Safety and comfort also can be ensured.***Budget requirements will have too be taken into account***Condusive environment is definitely a plus point, but not all places have such privileges. ***Yes of course but long way to go.***Although this is true, funding is a main issue. To begin with, target should be realistic. Rooms for group sessions can be used as meeting rooms as well. Must try to work on minimum budget so that other hospitals could obtain the same facilities.***Strongly agree****  Round 2 item: **Psychiatric appointment time should be at least 30 minutes for a follow up appointment and 90 minutes for a new patient appointment.**  Round 2 subclause change/ addition: *This includes the time needed for documentation, consultation from the specialist and discussion of the plan.* | Mean round 1: 3.9  Consensus reached |
|  | Round 1 item: **General comments on Resources**  *Service user/NGO comments: Agree on the consultation time, sometimes just 15 minutes consultation that make the patient feel frustrated.***Appreciate these points as a recovering patient. I'd love to contribute, and I'm sure many other recovering patients feel the same way. ***1.Introduce Peer Support Certification Program from other countries.*  *2.Actually more players can be involved in this collaborative work, including social entrepreneurs. Funding can come from other sources, not only government.*  *3.Make best use of existing resources, no need to spend so much to find new resources. Be creative and innovative. Think out of the box and sometimes we can learn from other countries/other sectors.***time is so important. Patients almost always feel that they are rushed and doctors don't give them the attention and care that they need. Hence its more like touch and go and you get a pill. ****  *Healthcare staff comments: ***Good ideas, but may need discussion with top management level regarding payment of honorarium due to budget issues.***Strongly agree**** | Consensus reached |
| 4.1 | Round 1 item: **The bureaucratic processes should encourage collaboration, rather than create barriers to collaboration (see appendix).**  *Bureaucratic barriers to collaboration include requirements to seek permission before collaborating with people outside the hospital and requirements for staff to fill a form on leaving the hospital.*  *Service user/ NGO comments: ***Processes needs to be there. We can't just allow people to do what they want. ****  *Healthcare staff comments: ***the current setting is seems bureaucratic within organisation as well as inter-agency collaboration. Some require a MOU to be signed.***Trust is missing by administrators for clinical staff***Ambiguity will arise when we work with Bomohs. Does this mean that we accept what they are doing? Does this mean that we from the scientific "community" agree with supernatural existence as what is practiced by Bomohs? Clear rules should be set before working with unregistered authorities. Because it can always backfire on us.****  Round 2 item: **The bureaucratic processes should encourage collaboration, rather than create barriers to collaboration.**  Round 2 subclause change/ addition: *Bureaucratic barriers to collaboration include requirements for staff to fill a form on leaving the hospital. The autonomy given to staff and teams needs to be determined individually, depending on skills, job requirements and experience.*  *Service user/NGO comments: Strongly agree***Ok***Its up to the patient and family members. It is their choice at the end of the day and we should be given them the choice. We do find many patients not having support post hospitalization and in the community. Bomohs are not the way to go despite many seeking their help. I think a lot of people are confused with what bomoh does (working with jins and black magic and witchcraft and such). What we need are correct spiritual healers that practice the word of GOD in place because with faith, with being grounded in religion is what will hinder a person from losing hope and choosing death plus it gives patients hopes and meaning in life. ****  *Healthcare staff comments: ***No additional comments.***Agree**** | Mean round 1: 4.5  IQD round 1: 0.5  Consensus reached |
| 4.2 | Round 1 item: **A ‘Friends of the Hospital’ group should be set up, together with a directory of services outside the hospital.**  *This will help form relationships with people outside the hospital to donate resources or services and lobby to improve resources available.*  *Service user/ NGO comments: Very true. Now the resources are scattered around and people have to search for them themselves, with their own effort. How good it is if we can unite all these resources in one place, so that people can easily access to them.***This would be awesome as this will greatly help patients to stay connected with the community and gives them a sense of hope knowing that there are people supporting them outside from the hospital . ***Excellent resource for relatedness. Other than that, it also helps to guide patients and families to the right sources.*  *Healthcare staff comments: ***a brilliant idea to involve public in care for persons with mental illness***can use the existing psychiatry visitors board***yes but not a high priority***Involve volunteers more***It is always good to have increased resources but one must be very clear that donation is a donation, it is a one way thing. Nothing personal should be expected in return.****  *Service user/NGO comments: Friend of the Hospital' is a brilliant idea***Ok***Sometimes I can't believe what professionals say. We need this because patients only get to see doctors once a month (if they are lucky), really briefly most of the time hence of course this list is really important. Mental health professionals must understand that without support it is nearly impossible for patients to recover. Hence why, this list is really important so that they feel brave and confident knowing that there are places and people to help and support them in the community. There are so many people doing so much of good work but most of the time we don't know whom and how to reach them. Having this in a list for patients would definitely be great and for doctors too. ****  *Healthcare staff comments: ***Good form of resource.***agree**** | Mean round 1: 4.4  IQD round 1: 0.5  Consensus reached |
| 4.3 | Round 1 item: **Specific mental health staff should form relationships with other people outside the hospital that help our patients**  *e.g. a specific specialist or other member of staff is responsible for providing training and support for a particular primary care clinic, school, or village.*  *Service user/ NGO comments: Very good! Mental health issue concerns the whole community, not just the patient alone. If training is provided to the outside community, the awareness can be raised and thus reducing the harm mental illness can cause to the patients and community.***Especially in schools, where mental illness is often taken lightly and school counselors do not help. ***Yes, this would be a great effort to support patients in the real world and to get them back on their feet. ****  *Healthcare staff comments: ***a community mental health nurse can play this role***Also with KOSPEN volunteer***Important***Mentor-mentee program***Training the trainers, wonderful idea, of significant impact. This must start from Day 1.Primary care personnels are the front liners. The community puts more trust in them than in people at tertiary care centres. So by training the front liners, target could be achieved more easily.****  *Service user/NGO comments: ***Ok***This is very crucial. We need mental health staff to engage with NGOS and recovering patients and carers, because who best to help patients than those with lived experienced and those that have first hand experience dealing with patients. Mental health professionals and staff should learn and listen because by having these insights you will understand more hence support better. We do find a lot of stigma amongst the doctors which is beyond belief sometimes. ****  *Healthcare staff comments: ***Strongly agree. Forming relationship with people in the community is important to reach out to patients and carers.***agree***Community based program can be established.**** | Mean round 1: 4.5  IQD round 1: 0.5  Consensus reached |
| 4.4 | Round 1 item: **Existing collaborative networks between primary care and people in the community should be used to help plan care for our patients (see appendix).**  *Service user/ NGO comments: This is good! But it needs extra effort from all stakeholders.***Yes I totally agree with this. This will definitely help patients in their recovery. ****  *Healthcare staff comments: ***especially here in Malaysia where people living in the same neighbourhood are quite closely knit***there is already well establish group (as mentioned above) thus facilitate the collaborative networks.***Invaluable, but difficult in practice***-****  *Service user/NGO comments: Ideal situation, hope can be done with all parties co-operation*  ****Leverage on technology. Can create app/online community to achieve this.***crucial****  *Healthcare staff comments: ***Strongly agree.***agree with comments***Of course, with patient's consent.**** | Mean round 1: 4.7  IQD round 1: 0.0  Consensus reached |
| 4.5 | Round 1 item: **Patients who are not directly under the psychiatric hospital, should be treated in primary care (rather than district hospitals).**  *Primary care clinics have existing collaborative networks with community members, such as village heads. It is also recommended by the World Health Organization and is more convenient for patients.*  *Service user/ NGO comments: The community should get involved rather than fold their arms and stand aside.***Some patient did not one to reveal their illness so better it is in hospital***This is a good idea. ***This would greatly benefit patients and help patients in their recovery. This is convenient for caregivers as well and makes them feel involved and well supported as caregivers too struggle and need help. ****  *Healthcare staff comments: ***we need to work more on this, and take advantage of the Kospen initiative by the government to involve the locals.***depending on the patient's condition***Availability of treatment in primary care clinics is helpful for patients with difficulty going to hospital, for example those who live in villages that are far from the hospital. Primary care doctors and staff are welcome to undergo training or attachment with psychiatry team to equip themselves with knowledge.***Primary care practitioners need to be engaged and trained***Probably primary care with FMS.***I encountered this issue when i did my research in rural areas of Sabah (Tambunan, Nabawan, Tenom). People in the community refused to participate in the questionnaire just because we were hospital staffs. When the village head came into the picture, I had 15 participants in total. This shows the power of the village head and the respect he has from the community. Hence, this is 1 of the most important relationship that has to be maintained.****  Round 2 item: **Patients who are not directly under the psychiatric hospital, should be given the option of being treated in primary care (rather than district hospitals).**  *Service user/NGO comments: Yes, the option must be given as not all patient want to reveal their illness***Ok****  *Healthcare staff comments: ***Agree that patients should be allowed the option.**** | Mean round 1: 4.3  IQD round 1: 0.5  2 respondents disagree  Mean round 2: 4.9  IQD round 2: 0.0  Consensus reached |
| 4.6 | Round 1 item: **First-responder training programs in mental health should be provided for other people that help our patients**  *(e.g. teachers, religious human resource staff)*  *Service user/ NGO comments: Very true! These people are at the front line compared to doctors and nurses. They are the ones who can prevent and detect mental health issues earlier.***Yes, not many have the adequate knowledge***This is crucial as we need to know how to help and what to do in time of crisis etc. We want to be able to help and do an informed decision than just trial an error. Every decision is crucial and must be taken seriously. ****  *Healthcare staff comments: ***strong evidence that this is very helpful namely in suicide prevention***Yes but the training module or program should be less technical term and include more lay terms***For example, mental health first aid and early warning signs of illness can be taught to others.***Crucial and ongoing***Mental Health First Aid programme is being recommended***similar to 5.2.1.2****  Round 2 subclause change/ addition: *e.g. teachers, religious leaders, human resource staff*  *Service user/NGO comments: Agree that Mental Health First Aid Programme should be implemented***Ok***we are waiting for this. We help patients 24/7 are in the community hence such trainings would be much appreciated. thank yoU!****  *Healthcare staff comments: ***Yes, strongly agree.***Agree, very useful in executing preventive measures**** | Mean round 1: 4.6  IQD round 1: 0.5  Consensus reached |
| 4.7 | Round 1 item: **Other people that help our patients need to know referral pathways and who to call if they are uncertain about what to do.**  *Service user/ NGO comments: This is good and it involves the whole community.***This is vital as not many people how receive psychiatric care (e.g not sure if they can walk in a psychiatric clinic or they need a referral from a psychiatric practitioner) ***This is crucial. They need all the help they can get. They have never gone through the illness and because psychological illness is something that can't be seen, it's hard for other people to understand and help. ****  *Healthcare staff comments: ***there should be a center of information on this (website etc)***yes agree***More information needs to be made available***Printed on their cards***Referral pathway is something we need to look into. At present, a patient has to go to KK, get a referral letter, go to psychiatry clinic, get an appointment and come back on that date. We need to look into how we can bypass this long pathway, or perhaps abolish appointment system for new cases.****  *Service user/NGO comments: Yes, referral pathway should be fine tune. I have heard one scenario, in which KK does not offer referral letter for the patient to Hospital (pcyhiatric) although the patient case is serious, suicidal. As a result of this, patient refused to seek help anymore due to frustration that she experienced***Need more outreach and publicity to increase awareness on how to help.***indeed****  *Healthcare staff comments: ***Yes, definitely agree.***Some psychiatry clinic also allow walk in case. Very important for them to know referral pathways in nearby clinics/hospitals**** | Mean round 1: 4.7  IQD round 1: 0.3  Consensus reached |
| 5.1 | Round 1 item: **Inviting to take part in problem solving and decision making.**  *Patients, carers and some staff may need to be explicitly asked if they would like to take part in the problem solving and decision-making process (see appendix below). There needs to be agreement on what the problem is that is trying to be solved.*  *Patients, carers and some staff sometimes make the assumption that they are not expected to take part in decision making. An explicit invitation reduces this assumption.*  *Service user/ NGO comments: ***Agree, all parties should be informed on this***I do feel that its important for patients and carers especially to feel that their opinion matter, because really they play a really crucial role in achieving this goal. By having an explicit invitation would give a positive impact indeed. ***As a patient, my default assumption (prior to this) would be that I only need to agree to what's given to me. So I'd feel more empowered when I know that I actually do have a part to play in problem-solving & decision-making.*  *Healthcare staff comments: Yes. They must be facilitated and empowered***This process can be conducted smoothly if the healthcare professionals were provided with standardized and appropriate materials during discussion eg flow charts/ pamphlets etc. This process may probably most effective in well educated patients, carers and some staff. Healthcare professionals should be well- informed about what must/ need to be told during the consultations. Difficulties may arise in those who have poor insight/ impaired judgement despite psychoeducation and longer time for consultations would be required.***yes, agree as we need to have more shared decision process in local setting (encourage on Person Center Care approach). However, for patients with mental health issue, I think their condition need to be take into consideration for involving them in decision-making.***Most of the time patients, carers and some staff may not be actively involved in problem solving or decision making process unless specifically asked.***Agreed***It is not asked "if they would like to take part", but to ask directly whatever needs to be solved and to be decided. Asking ' if they would like to take part' gives the option of 'not wanting to take part'. Due to the hierachial patient/carer - provider relationship, patient/carer fight shy of giving their opinion on problem solving, decision making. ***Decision making usually involves the treating doctor, patient and carer. Rarely staff will be invited.***I totally agree with these. However, the word invitation makes it sound optional. Problem solving, when it comes to treating or helping a patient, is not only the duty of a medical practitioner. The patient themselves and family members should take equal responsibility. Everyone in the treating team (doctors, nurses, community nurses) should be equally involved and ideas from each must be carefully discussed upon.***Totally agreed. This assumption arises from Malaysian hierarchical and paternalistic culture. Even when they are invited to participate in decision making , the lack of confidence to participate in them is observed. So, explicit invitation would gradually raise their confidence in taking position as important stakeholders in decision making.*  Round 2 item: **Inviting to take part in problem solving and decision making.**  Round 2 subclause change/ addition: *Patients, carers and some staff may need to be explicitly asked if they would like to take part in the problem solving and decision-making process (see appendix below).*  Round 2 appendix change/addition: *Patients, carers and some staff sometimes make the assumption that they are not expected to take part in decision making. An explicit invitation reduces this assumption. In patient consultations a simple invitational statement or question can be used:*  *"It sounds like the sleepiness is causing you lot of problems. Shall we think together about what we can do about it?"*  *Some patients and carers may not wish to take part in decision making and problem solving, particularly if they are feeling overwhelmed or if the treatment options are complex. For example, sometimes patients and families prefer their doctor to select the medication that they feel is best for them. In these cases, the practitioner needs to decide whether it is therapeutically appropriate to a) respect their wish and make the decision for them or b) further encourage the patient to be involved in making the decision.*  *Service user/NGO comments: Agree, to give option to participate based on the willingness of the patient to participate. Those who unable to make decision eg in the manic episode or too depressed, it is wise for the professional to decide what is the best for them (guided them through the process)***Agree. Caregiver/Patient must be given sufficient information to make an appropriate decision. Sometimes, their wish is made based on goggle search or hearsay from friends without fully understanding the whole picture***Agreed. If patients choose not to be involved, then they are the ones who must decide so (perhaps due to being overwhelmed, or other factors). Meaning that they use their rights to be helped in decision making, and not due to paternalistic practices or the automatic assumption that patients are unable to make decisions for themselves.***ok***I do feel that a shared decision must be made in all aspects because its the patients life and should be taken seriously and personally especially when it comes to medication and the side affects of it and serious therapy methods like ECT. Most patients feel disempowered because of how they are being treated and finally just follow whatever the doctor says despite horrible side affects etc. We must make patients understand that whatever they feel is important and to inform the doctors or family members immediately. Continuing medication with horrible side effects can lead to default in treatment and meds. When a patient is stable, the doctor and especially family members (carers) must ask them what would they want us to do when they relapse or become unstable. Do not wait until the patient is unstable to then ask them to make the decision because that becomes challenging in many instances. But I would encourage to still ask them first. ****  *Healthcare staff comments: Nil***Yes, i do agree sometimes assumptions are made regarding patients' and carers decision making process. This is a useful reminder to practice shared decision making between health professionals and patients or carers.***Patient and carers should always be invited for joint decision to decide on patient management. Depending on their preferences/ answers then a decision shall be made***Strongly agree with the new appendix. **** | Mean round 1: 4.7  IQD round 1: 0.5  Consensus reached |
| 5.2 | Round 1 item: **Identifying stakeholders.**  *The people that are either affected by the problem or could help with the problem need to be identified (see appendix below). Decision making needs to be done at an optimal time, with these people present if possible. If they cannot be present at the time of decision making, then knowledge, opinions and concerns should be asked from them first.*  *People affected by the decision normally include the patient and family. People that may help with the problem include patient, family, healthcare staff and other people in the community, such as employers, teachers and community leaders. If healthcare staff have been previously involved and know the patient well, then their opinion should be asked before important decisions are made.*  *Service user/ NGO comments: ***Indeed. Important decisions cannot be rushed. We must have all the input before making an informed decision. But we can't have too many people representing a party because that would be messy and lengthens the decision making process. ****  *Healthcare staff comments: ***A regular meeting with appropriate agencies should be arrange to discuss on the progress of patients w/o compromising the confidentiality.***Yes, but this need to be done with consideration of the patients. How comfortable of patients with the present of others beside healthcare provider and family members***May be subjected tho time constraints***Due to stigma issue and sensitivity of mental health problem, people involved should be restricted to those who really need to know, with the consent of caregiver or patient. ***Very true but the decision making process will be delayed in order to get all the parties involved on board. Probably not suitable for some issues that need relatively urgent decision making***Even if the healthcare staff was not involved or does not know the patient well previously, their opinion should also be asked because they might have similar (or even better) experience in treating other patients with similar issues.***Agreed*  Round 2 item: **Identifying stakeholders.**  Round 2 subclause change/ addition: *The people that are either affected by the problem or could help with the problem need to be identified (see appendix below). Decision making needs to be done at an optimal time, with these people present if possible. If they cannot be present at the time of decision making, then knowledge, opinions and concerns should be asked from them first. This should be done with the patient's agreement (see appendix).*  Round 2 appendix change/addition: *People affected by the decision normally include the patient and family. People that may help with the problem include patient, family, healthcare staff and other people in the community, such as employers, teachers and community leaders. If healthcare staff have been previously involved and know the patient well, then their opinion should be asked before important decisions are made. Contacting other people must be done with the permission of the patient, including contacting family members. If the patient does not have capacity to make the decision about involving other people, then the decision to contact them should be made in the best interest of the patient, taking into consideration the balance of risk, the opinions of other people close to the patient, legal requirements and any advanced directives. In many cases it is appropriate to just ask the patient about the opinions of other people, eg "What does your family think about you taking medication?, "Are their people in your family who do not think it is a good idea?", "What do you think about your aunt's belief that taking medication will cause kidney problems?".*  *Service user/NGO comments: Strongly agree on this.****  *Agree***Agreed, but with some reservations on the last paragraph. As a patient, sometimes I'd have problems telling objectively about the opinions of other people. For example, I might not realize that my family objects me from taking certain medications due to appropriate reasons. I suggest families to be contacted so they could discuss their perspectives with the practitioners. ***ok***I stick to important decisions cant be rushed. We must be patient especially being family members. I do feel that in the Malaysia context we like to rush in making decisions hence why medications becomes a must nowadays treating patients. Give a patient a cup of tea, a smile and your time. It will get better. ****  *Healthcare staff comments: Nil***Agree, patient should give consent for health professionals to cantact relevant people. However, in emergency situations this may not be suitable.***good***Agree***Very well explained.***agree**** | Mean round 1: 4.7  IQD round 1: 0.4  Consensus reached |
| 5.3 | Round 1 item:  Round 2 item: **Defining the problem.**  Round 2 subclause change/ addition: *The problem needs to be clearly defined and agreed upon.*  Round 2 appendix change/addition: *In patient consultations the problem this may first involve exploring the patient's goals or values and working out the barriers to reaching these goals or following values.* | Mean round 2: 4.8  IQD round 2: 0.0  Consensus reached |
| 5.4 | Round 1 item:  Round 2 item: **Finding common goals and values.**  Round 2 subclause change/ addition: *Common goals or values in solving the problem should be defined and agreed upon.*  Round 2 appendix change/addition: *Goals involve a defined end point (e.g. finding a job), values give a general direction (e.g. would like to contribute). Goals should be found that all parties would like to work towards.*  *Service user/NGO comments: ***agree***Very good.****  *Healthcare staff comments: None***Patients and carers should have a clear idea on what the problem is and what are the goals for problem solving. This would avoid resentment and ensure better compliance.***clear goals will help every parties to work better with each others and help to reflect is something goes wrong even at early stage***Include brief steps to adhere in case consensus is not achieved.**** | Mean round 2: 4.8  IQD round 2: 0.0  Consensus reached |
| 5.5 | Round 1 item: **Sharing of knowledge, opinions and concerns.**  *Knowledge, opinions and concerns, which will help with problem solving should be shared between the people present.*  *Service user/ NGO comments: ***This is will help in making an informed decision****  *Healthcare staff comments: ***Getting the information out msy be a problem***Due to stigma issue and sensitivity of mental health problem, people involved should be restricted to those who really need to know, with the consent of caregiver or patient. ***As mentioned earlier, a few of the people involved in solving the problem might not be present. So, it must be shared with them too, not only with the ones who are present.***This is to understand different perspectives/needs and reduce misunderstandings that will facilitate achievement of treatment goals for the patients while supporting others in their issues.*  Round 2 appendix change/addition: *This process can involve a formal problem solving technique, starting with a brainstorming, where all possible ways of solving the problem are written down and the pros and cons of each solution discussed.*  *This process may need to involve people outside the meeting, in which case it may be necessary to defer decision making until all parties have shared information, opinions and concerns. For example, a patient may wish to defer a decision about switching medication until the next appointment, after sharing information about a new medication with family members and finding out their opinions.*  *Service user/NGO comments: I agree with this***Formal meeting to solve a problem need not involve everyone but key person. Should not be too many, or else it becomes too cumbersome to make a simple decision. ***Agreed***ok***Yupp agreed. thanks****  *Healthcare staff comments: None***I find that the example described in the new appendix is relevant to Malaysia's society - quite often I meet patients who would like to discuss their treatment options with their family members.***Yes, patients whose judgment is intact and insight is good should be given more time to make any decision. Small notes- as long as it doesn't cause any further harm to patient or others.***Agree on modified content***yes**** | Mean round 1: 4.6  IQD round 1: 0.4  Consensus reached |
| 5.6 | Round 1 item: **Making the final decision.**  *All opinions should be taken into account when making the final decision, including opinions of people not physically present or not able to make decisions.*  *Service user/ NGO comments: ***Yes as we want a consensus ***What if the patient himself/herself is not able to make the decision due to reasons such as mania? Each stakeholder that makes a decision must be able to make decisions voluntarily.***Yes, because the opinion of the person that's absent might be the most important. Hence, we should not eliminate everyone. ****  *Healthcare staff comments: ***When making decisions related to the care of patient, all relevant parties and resources should be considered. For example, in the continuation of care for patients in the community, are there sufficient resources to help family with this. The representative in the community, such as other villagers, or extended family members may not be able to be present during a meeting. However, their opinions should also be considered.***Subjected to constraints in the real world***-***Getting opinions of people not physically present or not able to make decisions is time consuming, therefore possibly hampering efficient decision making process.***The opinion of all affected by a decision should be taken into account to ensure smooth implementation of the decision.*  Round 2 item: **Making the final plan.**  Round 2 appendix change/addition: *Patients may not have the capacity to make a decision at the point the decision has been made. However, many patients will have discussed what they want to be done in the event of future relapse. This may be in the form of a formal advance directive or relapse plan or they may have informally discussed this with a relative or member of the healthcare team. An example of this is a patient who writes a relapse plan which requests ECT in the event of becoming manic. This request would be considered by the healthcare team, when making decisions about ECT.*  *The amount of time taken in taking opinions from different people needs to be proportionate to the importance of the decision, who else the decision will affect, the urgency in making the decision and the resources available. For example, more time will be spent asking different opinions about the decision to discharge a high risk patient than would be spent asking opinions about which medication to choose.*  *Service user/NGO comments: ***bearing in mind that the last paragraph of new appendix***cannot consider all opinions from those who are not present. I believe in doctor's expertise to make the best decision.***If the person that is not physically present does carry so much weight, like a brother that lives in another state, then we can proceed with the decision just by calling and asking his opinion. But we don't have to wait for him to be physically present to make that decision. ****  *Healthcare staff comments: ***The new appendix takes into account the issues raised regarding this matter. ****  Round 3 item: **Making the final plan.** *Changed to: After deliberating the options, the plan should be produced. This should be written down for the patient if it is complex. The opinions of people not physically present or not capable of decision making at the time shoud be considered if they* | Mean round 1: 4.4  IQD round 1: 0.4  2 respondents disagree  Mean round 2: 4.5  IQD round 2: 0.5  1 respondents disagree  Mean round 3: 4.6IQD round 3:  Interquartile deviation round 3: 0.5 |
| 5.7 | Round 1 item: **Implementing the decision and making clear that the decision can be reviewed.**  *Discussion should take place about who should do what. A review date should be set. It should be made clear to patients and carers that the decision can be reviewed if the solution is not effective or makes the problem worse.*  *Service user/ NGO comments: ***It's a good learning process for all parties. ****  *Healthcare staff comments: ***Possible difficulties: commitment from other agencies. ***I think it is helpful that we can set a clear plan to patient and carers, and what to do next should Plan A does not work.***Sometimes decision may change depending on new findings, evidences etc***review date is of great importance. If no dateline is given, nothing will move forward.***Perspectives and needs are dynamic in nature contributed by many factors, therefore, a review process is important to ensure smooth implementation of a decision. However, it may delay the process. A cycle of decision making - implementation- review and decision-making - implementation, would be useful for longer-lasting improvements.*  *Service user/NGO comments: I strongly agree that decision can be review to tailor to the needs of patient as it is not black and white situation as many factors could interfere with the recovery process.*  *If the formal decision is not working, it can be review and given another option***agree***ok****  *Healthcare staff comments: None***No further comments.***agree**** | Mean round 1: 4.8  IQD round 1: 0.0  Consensus reached |
|  | Round 1 item: **General comments on 'Collaborating with people outside the hospital'**  *Service user/ NGO comments: This is currently lacking in the mental healthcare system. The outsiders should not just fold their arms and pretend they are not related to this. In fact, this concerns everyone. I believe there are many people who care and willing to help outside there. Someone has to take initiative and combine all these forces together.***Suggest to patient any activities related to mental health, such as NGOs talk about mental health careers. ***The doctors and hosp staff must be open to this idea and must realize that everyone has their own area of expertise and knowledge. Sometimes because doctors and those from the medical field feel or regard themselves as the 'experts' it may hinder from us achieving this goal as they might not want to listen or take in the opinion of others. ****  *Healthcare staff comments: ***Outreach programs such as mental health awareness program, forum, or health exhibition done in the villages, school, public hall or other agencies in the community.***Resources of all forms need to be provided fit the above to be possible***Direct link should be given to people in the community to reach psychiatry personnel. ***Specific training for community based care****  *Service user/NGO comments: All have been mentioned above***Collaborate with NGOs to reach the community, eg the masjid, temples, schools, hobbies clubs, etc. Mention in the community program / talk that the hospital invites the community to collaborate, that mental health is everybody's business. Not just the hospital's, the patient's, or the family's. Understand what the motivation of these groups are, and appeal to it. Eg: Muslims would respond to calls that align with the verses in the Quran / hadith. For example, Muslims believe that it's important to help each other in calamity, because that way God will help them in return in the Hereafter. So understand what motivates them.***Be open to this and do realize that we are students for life and there's more that we don't know. There are doctors unfortunately that feel they know enough hence why this collaboration will be challenging to work. Let's understand that we can't know everything and that people with lived experience and carers would have greater insights and experience that will be of great help. ****  *Healthcare staff comments: ***No additional comments.***Mental health program should be part of module in education system, social welfare etc***Involvement of higher authorities is also important**** |  |
| Delphi a | Round 1 item: **I understand the rationale for the Delphi process** | Mean round 1: 4.5 |
| Delphi b | Round 1 item: **Filing the form was easy** | Mean round 1: 3.9 |
| Delphi c | Round 1 item: **Filling the form took longer than I expected** | Mean round 1: 2.6 |
| Delphi d | Round 1 item: **The Delphi process is a useful way to make new guidelines** | Mean round 1: 4.1 |
|  | Round 1 item:  **Please write any other comments about these guidelines or suggest any other items**  *Service user/ NGO comments: Many people do care but they are not sure where and how to start. Hopefully something can start soon.***I think most of the items are good and ideal ***Psychiatry collaborative practice should be at par as other medical field. It should not be treated as a less important department in hospitals or community. As much as other chronic illnesses that had been educated to the public, mental illnesses should be taken into consideration too. ***Advocacy and Patient Empowerment should be highlighted as a part of the Recovery Plan. The goal should be recovery but also to promote awareness, reduce stigma, stop discrimination and achieve equality in the workplace, universities, schools, home etc. Because when all this happens then can only the much needed change happen. Humanising the mental health care in Malaysia is so important for one's recovery. Yes, we are pushing for this collaborative effort, but what next. What's the impact like and when are we expecting to achieve this. We need a time line to achieve all this. ****  *Healthcare staff comments: ***The guidelines have covered relevant and important areas of collaborative practice.***I agree with the theory of tjis but worry about the actual implementation due to resource limitation***It would be useful to specify in the beginning, the context or situations on which this Delphi process is to address. Many of the statements posted are too generic and theoretical/academic in nature, and hence the responses are very general as well. The situations must also take into consideration healthcare system in Malaysia, which is unlike developed countries where social support system and government support are very strong. Over in Malaysia, the government provides no financial support for NGO mental healthcare service. Hence, their role is very limited, and almost non-existent in areas outside the Klang Valley and a few big cities.***FMS should be included in this guidelines***School counselors could play a major role in mental health issues. After all, early education could lead to prevention, which is always better than cure. ***Awareness program within community is equally important before starting collaborative work with them.****  ***Round 2***  *Service user/NGO comments: This is am ideal state, I hope bit by bit we can implemented it in real setting. If after the implementation, some of it is found to be ineffective, it should be revised. ***Excellent effort! May be idealistic to some people, but I think it is much much better than doing nothing and expecting miracles to happen. Instead of cursing the darkness why not we become the light? What [research team] came out with is definitely a good start to roll out a road map to a better mental healthcare system in Malaysia. I support 200%. Let me know how much more I can help, and I will help if I can.***More advocacy work and patient empowerment is needed. We need to highlight more about inclusion and choice. Especially in the context of Malaysia, 'gatekeepers' being the doctors. We need doctors to take a step back and let patients also decide as it is their life we are talking about, discussing about and making decisions on. Mutual respect and getting patients and carers involve is crucial in ensuring such an effort is successful. Stigma is still the biggest barrier to mental health and unless education and awareness work is aggressively being done, things will never change. I am really grateful that you guys are considering such an effort because this is much needed. ****  *Healthcare staff comments: ***All items are good and what we would like to achieve in providing the best mental health care. Hopefully all these can become a reality in the near future.***agree***Establishing standardized steps/procedures will help collaborative process much easier.**** |  |
|  | Round 1 item: **Comments about this process**  *Service user/ NGO comments: This is good as you care about opinion from all people. But I feel the ratio of patients and caregivers is underrepresented, with too many psychiatrists and doctors. Perhaps you can find more people for this category?***It took me quite a long time to understand the term used as I am a patient, not a staff. Some of the question is not related to me as I never know the SOP in the hospital.*  *Maybe there should be separated questionnaire for the staff and for the patient or careers/community to make it relevan for us to answer it.*  *Thank you.***This is such a great effort. Filling out this form made me feel closer with everyone involved despite the fact that I haven't met probably half of the people in this committee. I also feel appreciated, that our voices matter and that a change will be made and that's indeed progress! Thank you so much for this and I look forward to the next one. Thank you. ****  *Healthcare staff comments: ***for statement on 'useful way to make new guideline' - I am not sure if one round of delphi process is sufficient to reach the consensus? ***Interesting***A short introdution on mental healthcare system in the country and gaps that needs addressing is needed to set the context on which this Delphi process trys to address. In other words, what is the problem that needs fixing in this study and recommendations. ***General ideas and suggestions are helpful for effective patient management and care plan.****  ***Round 2***  *Service user/NGO comments: I am grateful that this effort are make for the betterment of mental health patient and mental health management as a whole***I truly appreciate the opportunity to contribute my perspective on this process. I hope this will bring the much-needed change in our country. I understand it's just one of the many efforts done by the HCP. As a patient and user of the service, I hope to see others to receive better quality and more holistic treatment. Thank you for doing this, may God reward and bless you.*  *I'd like to suggest one last thing - that recovery to be emphasized early on in the process of treatment. ***This is my first time getting involved in a Delphi process. I feel I am involved in nation-building and we are all working towards a better Malaysia, better society and better standard of living. Process is long but it is unavoidable. It is good that you give us a reasonable timeframe to allow us to take part according to our pace. Thank you so much!***Thank you so much. I do feel that this is a great way to get our voices heard. However, the mix is not balanced hence the answers will always lean to a medical model rather than a social model and will again fall back to what the mental health professionals feel, think and want and do not fully represent what the patients and carers fully need and want. However, this is a great start and we are really appreciative of such an effort as we do appreciate how much work and effort it has taken in trying to do this. Thank you, thank you and thank you!****  *Healthcare staff comments: ***It was a good opportunity to learn from other professionals as well as patients and caregivers. **** |  |
|  | Round 1 item: **Have you ever taken part in a Delphi process before?**  ***No***No***No***No***No***No***No***No***No***  No***No***No***No***No***No |  |
